# Father participation in the care of a critically ill child: a scoping review

**DOI:** 10.1590/1980-220X-REEUSP-2024-0278en

**Published:** 2025-04-28

**Authors:** Rafaela Sterza da Silva, Cinara Bozolan Coppo, Edrian Maruyama Zani, Adriana Valongo Zani Zani

**Affiliations:** 1 Universidade Estadual de Londrina, Programa de Pós-graduação em Enfermagem, Londrina, PR, Brazil.

**Keywords:** Fathers, Father-Child Relations, Paternal Behavior, Child Care, Catastrophic Illness

## Abstract

**Objective:**

To map and describe existing studies in the literature on fathers participation in caring for critically ill children.

**Method:**

Scoping review according to the JBI methodology. The search took place in October 2023, with studies published in full in Portuguese, English or Spanish, without time limits and indexed in databases and portals of theses and dissertations. Two reviewers read the title and abstract of the studies and exported the data to a Microsoft Excel® spreadsheet for grouping and removing duplicates. Data synthesis was done in written and visual form, using diagrams.

**Results:**

The search resulted in 961 studies, with 38 being eligible to compose the sample. The studies analysis allowed us to find that paternal participation in the care of a critically ill child involved benefits for the child, the mother, and the family; the forms of paternal care; the father’s social role; the psycho-emotional, socioeconomic, and family impacts; and gender and sociocultural barriers regarding the paternal figure.

**Conclusion:**

Paternal participation in caring for a critically ill child has proven to be a complex phenomenon that requires the deconstruction of historical, sociocultural, and gender stigmas related to parental roles.

## INTRODUCTION

Due to political, economic, social, and religious influences, the family unit has undergone major transformations over the years in terms of its concept, structure, dynamics, and role played by each member. The family is understood as the basis for the organization of a society and, for centuries, it was founded on patriarchy, where women had no voice and their only role was to take care of the house and raise children, while men were responsible for the role of authority and family provision^(1)^.

The father’s role in contemporary society has evolved significantly, going beyond the role of mere financial provider and family authority. Today, fathers are increasingly involved in the care and education of their children, playing an active role in transmitting values, affection, and protection^(2)^. This transformation in fatherhood is reflected in the redefinition of the concept of father, who now sees himself as the protagonist in raising children, seeking to establish deep emotional bonds and actively participate in the family’s daily life^(3,4)^

However, society still carries traditional patterns that often relegate the father to a secondary role in raising children, especially in caregiving contexts. The father figure, despite his desire for greater involvement, faces cultural and social obstacles that associate parenthood mainly with the maternal figure^(5,6)^. This traditional view limits paternal involvement, making many fathers feel like they are supporting actors in the process of raising and caring for their children^(7)^

This duality of roles is exacerbated in situations of childhood illness, where the paternal figure tends to return to his role as family provider, reflecting the conflict between the modern conception of paternity and the retrograde ideology of masculinity. In this context, many fathers still feel pressured to assume the family’s financial responsibilities, while direct care for the sick child is centered on mothers, perpetuating gender stigmas rooted in society^(8,9)^

Scientific literature highlights the importance of paternal involvement in child care, especially in cases of serious illness. Studies show that the father active participation in caring for a sick child can has a positive impact on the child’s development and improves the quality of care provided^(10,11)^. However, the active participation of the father is not a consensus among scholars, showing that many fathers still operate under traditional gender concepts, limiting the exercise of paternity^(12)^.

Although father involvement in the care of a sick child is controversial, the literature also does not offer a precise definition of serious childhood illness. However, it establishes a list of illnesses characterized by their progressive and long-lasting development, with the potential to cause disabilities, dependence on care and threat of death, highlighting the complexity of conditions that require continuous and intensive care^(13)^. In the context of this study, serious illness was defined as a clinical condition with the potential to cause serious health consequences, which may lead to significant disability, severe complications, or even death. Typically, these diseases require prolonged hospitalizations due to the need for numerous medical interventions.

During these children’s treatment and rehabilitation, paternal participation is often minimal or even absent, highlighting the urgent need for greater awareness raising and support to promote equity in parental involvement in the care of a critically ill child^(14)^.

Studies demonstrate advances in knowledge about paternity, sociocultural and gender influence on the exercise of paternity, paternal role in the father-child relationship, and the paternal function in child development, evidencing recent interest on the part of researchers in the paternal figure^(15,16,17,18,19,20,21)^. However, little is known about the exercise of parenthood in the face of a critically ill child. In a survey in *Open Science Framework and* in the *International prospective register of systematic reviews* (PROSPERO), no existing scoping reviews or systematic reviews were identified that answer the question: how does the father participate in the care of a critically ill child?

Due to the scenario presented, with the aim of revealing the paternal figure and contributing to the consolidation of knowledge about paternal care for a critically ill child, the objective of this study was to map and describe the studies in the literature on the participation of the father in the care of a critically ill child.

## METHOD

### Design of Study

This is a scoping review that sought to explore, map, synthesize scientific evidence and identify knowledge gaps regarding the presence and participation of the father in the care of a critically ill child. The present study was developed in accordance with the JBI manual^(22)^ and the guidelines of the *Preferred Reporting Items for Systematic Reviews and Meta-Analyses extension for Scoping Reviews* (PRISMA-ScR) *Checklist and Explanation* for transparency in the drafting of the review report^(23)^

The protocol for this review was registered in the *Open Science Framework* (OSF), under the identification https://osf.io/5fzrg/DOI 10.17605/OSF.IO/5FZRG

To prepare this review, five steps were followed^(24)^: 1) identification of the research question; 2) identification of relevant studies; 3) selection and initial evaluation of studies; 4) data analysis; and 5) grouping, synthesis, and presentation of data.

### Data Collection

To formulate the research question (stage 1), the mnemonic PCC (Population, Concept and Context) was used, described in chart 1.

**Chart 1:** PCC Strategy (Population, Concept and Context) – Londrina, PR, Brazil, 2024.

	PCC	Description
P	Population	Father
C	Concept	Studies addressing paternal care
C	Context	Child care, Serious or catastrophic illness

Based on this strategy, the research question was developed: How does father participation in the care of a critically ill child take place?

The identification of relevant studies (step 2) involved the selection of databases, definition of search strategies based on descriptors and Boolean operators, and the formulation of inclusion and exclusion criteria. The search was carried out in the following databases: CINAHL (*Cumulative Index to Nursing and Allied Health Literature*), Embase (*Excerpta Medica dataBASE*), *Scopus*, *Web of Science*, SciELO (*Scientific Electronic Library Online*), PubMed and the Virtual Health Library (VHL). For investigation of grey literature, the following was used: *Google Scholar* and the Catalog of Theses and Dissertations of the Coordination for the Improvement of Higher Education Personnel (CAPES). The recovery of studies in *Google Scholar* was carried out on the first 30 (thirty) pages. It should be noted that the search strategies were adjusted in accordance with the particularities of each database.

Data collection took place in October 2023 through the journal portal of the Coordination for the Improvement of Higher Education Personnel (CAPES), via the Federated Academic Community (CAFe). The “advanced search” feature was used with MeSH (*Medical Subject Headings*) and DeCS (Health Sciences Descriptors) descriptors, and boolean operators *AND* and *OR*. In the CAPES Catalog of Theses and Dissertations, the search strategy of the solitary term and the crossing of terms without Boolean operators was applied. The strategy involved crossing the terms among them, as shown below:

- Health Sciences Descriptors (DeCS): Pai *AND* Relações Pai-Filho *OR* Comportamento Paterno *AND* Cuidado da Criança *AND* Doença Grave.
- *Medical Subject Headings* (MeSH): *Fathers AND Father-Child Relations OR Paternal Behavior AND Child Care AND Critical Illness.*

The uncontrolled descriptors “sick child” and “father’s care” were incorporated. Below, in Chart 2, the search strategy used in each database is detailed.

**Chart 2:** Development of the search strategy in the various databases/portals – Londrina, PR, Brazil, 2024.

Database	Search strategy
VHL	#1 Pai *AND* Relações Pai-Filho *OR* Comportamento Paterno *AND* Cuidado da Criança *AND* Doença Grave; #2 Pai *AND* Cuidado da Criança *AND* Doença Grave; #3 Pai *AND* Cuidado da Criança *AND* “Filho doente”; #4 Comportamento Paterno *AND* Filho Doente *AND* Doença Grave
CINAHL	#1 *Fathers AND Father-Child Relations OR Paternal Behavior AND Child Care AND Critical Illness*; #2 *Fathers AND Child Care AND Critical Illness*
PubMed	#1 *Fathers AND Father-Child Relations AND Child Care AND Critical Illness*; #2 *Fathers AND Child Care AND Critical Illness*; #3 *Paternal Behavior AND Child Care AND Critical Illness*; #4 *Paternal Behavior AND “Sick Child” AND Critical Illness*; #5 *Paternal Behavior AND “Sick Child”*
Embase	#1 *Father AND Father child relation OR Paternal Behavior AND Child Care AND Critical Illness*; #2 *Father AND Child Care AND Critical Illness*; #3 *Father child relation OR Paternal Behavior AND Child Care AND “Sick Child”*; #4 *Father child relation OR Paternal Behavior AND “Sick Child”*; #5 *Father AND Child Care*
Scopus	#1 *Fathers AND Father-Child Relations AND Child Care AND Critical Illness*; #2 *Father AND Child Care AND Critical Illness*; #3 *Paternal Behavior AND “Sick Child”*
*Web of Science*	#1 *Fathers AND Father-Child Relations OR Paternal Behavior AND Child Care AND Critical Illness*; #2 *Fathers AND Child Care AND Critical Illness*; #3 *Paternal Behavior AND “Sick Child”*; #4 *Fathers AND Child Care AND “Sick Child”*
*SciELO*	#1 *Fathers AND Father-child relation OR Paternal Behavior AND Child Care AND Critical Illness*; #2 *Fathers AND Child Care AND Critical Illness*; #3 *Father-child relation AND “Sick Child*”; #4 *Fathers AND “Sick Child” AND Critical Illness*; #5 *Fathers AND Child Care*; #6 *Fathers AND “Sick Child”*
Google Scholar	#1 Pai *AND* Relações Pai-Filho *OR* Comportamento Paterno *AND* Cuidado da Criança *AND* Doença Grave
CAPES	#1 “Cuidado do Pai”; #2 “Filho Doente”; #3 Relações Pai-Filho; #4 Comportamento Paterno

### Selection Criteria

The eligibility criteria for the studies were established to ensure that only documents relevant to the scope of the review are included in the analysis, ensuring the obtainment of a high-quality review, supported by relevant evidence on the presence and participation of the father in the care of the critically ill child.

This review included studies published in established databases and gray literature that involved the father as a population and addressed the concept of paternal behavior, father-child relationship, and paternal participation in child care in the context of serious or catastrophic illness.

Thus, the following eligibility criteria were established: publications without a time frame, in Portuguese, English, and Spanish, of any methodological design, available in full and free of charge in electronic media, as well as dissertations and theses, which answered the research question. Studies of the editorial type, response letters, opinion articles were excluded, and duplicate documents were counted only once.

### Data Extraction and Analysis

The initial selection and evaluation of the studies (stage 3) involved the participation of two independent reviewers who, preliminarily, carried out an accurate reading of the title and abstract of the studies found. Then, data from eligible studies were exported to a *Microsoft Excel®* spreadsheet, so that the grouping and removal of duplicates could take place. To conclude, the reviewers read the materials in full, applying the eligibility criteria once again for subsequent data extraction.

Citation research was carried out on all studies that met the inclusion criteria to find additional studies. The studies chosen for this new investigation were analyzed in the same way as the others, as mentioned above, and identified separately in the results presentation.

Disagreements within researchers during the study selection process were resolved by a third reviewer, with expertise in the subject, who provided the final opinion. The quantitative results of each database, excluded studies and their reasons, total number of studies included for data analysis and synthesis were described by means of a flow diagram0

Data analysis (stage 4) involved the analysis and categorization of the extracted data with the aim of identifying patterns, trends, and gaps in the literature. The studies were analyzed by two independent authors and a synoptic table, prepared by the reviewers, served to systematize the extracted data, which consisted of the following information: study identification, authors’ names, year of publication, title, type of publication, country of origin, journal, type of study, objective of the study, and main results and considerations.

It should be noted that the synoptic table for data collection was previously evaluated by the authors through a pilot test. The pilot test for data extraction was employed by two researchers, who used the same search strategy mentioned above and tested the synoptic table in 10 studies randomly to align the eligibility criteria and ensure that all relevant data were covered. After the pilot search in the databases and testing carried out on the synoptic table, the two researchers responsible for the pilot test met with the other authors to discuss and evaluate the applicability of the synoptic table, the search strategy and whether the methodology used made it possible to achieve the research objectives. It is worth noting that, after discussion among researchers, there was no need to reformulate the data search and extraction process.

The grouping, synthesis, and presentation of data (stage 5) occurred in written and visual form, using diagrams, with the purpose of creating visualization and understanding of the results. Accordingly, a critical analysis of the results was carried out, presenting a descriptive and reflective analysis of the evidence obtained.

### Ethical Aspects

The research was carried out in accordance with ethical principles and applicable methodological guidelines, complying with good practices in scientific research and respecting the copyright of the studies cited. As this is a review study based on the analysis of publicly available secondary data, and as it does not directly involve the participation of human beings, there were no ethical issues requiring the assessment of an ethics committee.

## RESULTS

The search resulted in 961 studies, with 135 publications removed due to duplication. Thus, 826 studies were analyzed based on the title and abstract, with 694 documents excluded for not addressing the theme, due to the unavailability of the full text and because they were review studies, leaving 132 documents for full evaluation regarding the eligibility criteria. Of these, 96 studies were excluded, resulting in the selection of 36 documents. It is worth noting that two publications were added from the list of references, totaling 38 studies to compose the final sample.

**Figure 1:**
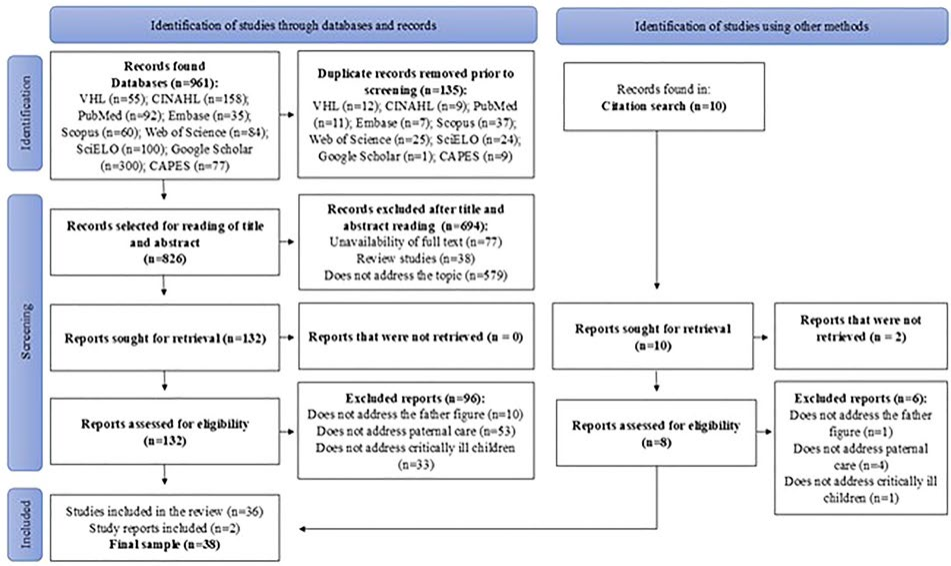
Study selection flowchart adapted from PRISMA-ScR^(25)^ – Londrina, PR, Brazil, 2024.

Figure 1 illustrates the flow diagram of the study selection process according to JBI recommendations, adapted from *Preferred Reporting Items for Systematic reviews and Meta-Analyses extension for Scoping Reviews Checklist* (PRISMA-ScR)^(25)^.

**Figure 2:**
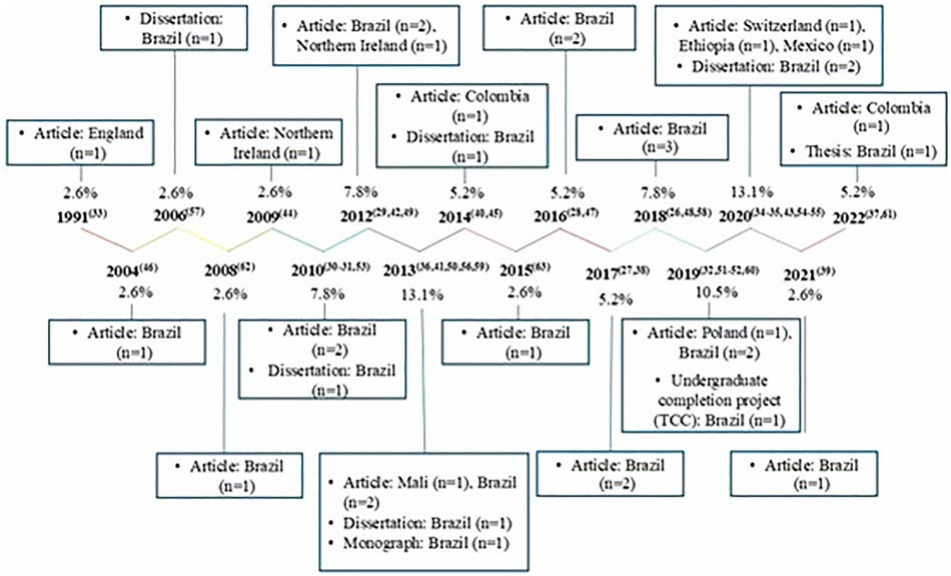
Timeline of selected studies according to year of publication, type of publication, number of publications according to country of origin and percentage of publication according to year – Londrina, PR, Brazil, 2024.

The 38 studies selected to compose the results of this research were categorized and organized in a linear sequence of increasing time, according to the year of publication, type of publication, country of origin, and database (Figure 2). It should be noted that the citations in figure 2 were numbered sequentially according to the order of the databases used in the search strategy, with VHL^(26,27)^, CINAHL^(28–31)^, Embase^(32,33,34,35)^, PubMed^(36)^, SciELO^(37,38,39,40,41)^, Scopus^(42)^, *Web of Science* ^(43,44)^, CAPES^(45)^, *Google Scholar* ^(46,47,48,49,50,51,52,53,54,55,56,57,58,59,60,61)^, and search for citations/other sources^(62,63)^.

The years of greatest scientific production on the subject were 2013^(36,41,50,56,59)^ and 2020^(34,35,43,54,55)^, totaling five studies each, followed by the years 2019^(32,51,52,60)^ (n = 4), 2010^(30,31,53)^, 2012^(29,42,49)^, and 2018^(26,48,58)^ with three publications each; 2014^(40,45)^, 2016^(28,47)^, 2017^(27,38)^, and 2022^(37,61)^ with two studies each year. The years with only one publication were 1991^(33)^, 2004^(46)^, 2006^(57)^, 2008^(62)^, 2009^(44)^, 2015^(63)^, and 2021^(39)^.

The majority of publications were scientific articles^(26,27,28,29,30,31,32,33,34,35,36,37,38,39,40,41,42,43,44,46 47,48,49,50,51,52,58,62,63)^ (n = 29). The dissertations^(45,53,54,55,56,57)^ represented 15.7% of the studies and there was only one undergraduate course completion project^(60)^ (TCC), one monograph^(59)^, and one thesis^(61)^.

The studies’ countries of origin were varied, with Brazil being^(26,27,28,29,30,31,38,39,40,41,42,,46,47,48,,50,51,52,53,54,55,56,57,58,59,60,61,62,63)^ the country developing the greatest number of studies on the subject (n = 28), followed by Northern Ireland^(44,49)^ (n = 2), Colombia^(37,45)^ (n = 2), England^(33)^ (n = 1), Switzerland^(34)^ (n = 1), Ethiopia^(35)^ (n = 1), Mexico^(43)^ (n = 1), Mali^(36)^ (n = 1), and Poland^(32)^ (n = 1).

The analysis of the studies allowed observing that paternal participation in caring for a critically ill child involves three categories: 1. Father participation in caring for a critically ill child, which discusses the benefits of paternal participation, the ways of caring, and the paternal role with the sick child, with the family unit, and with the mother; 2. Repercussions of having a sick child, which elucidates the psycho-emotional, socioeconomic, and family impacts experienced by the father; 3. Obstacles to paternal care, which portrays gender and sociocultural barriers regarding the father figure.

**Figure 3:**
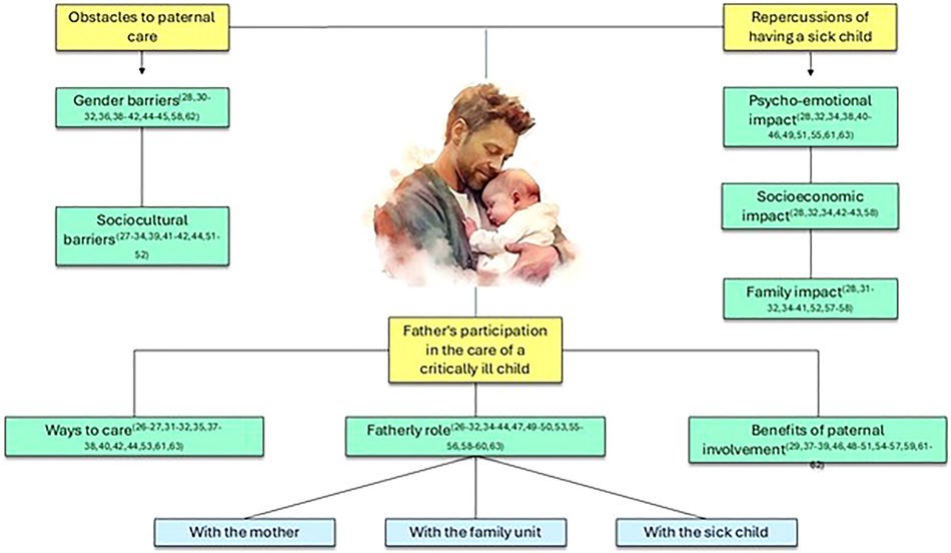
Mind map of the main results of the studies included in the scoping review – Londrina, PR, Brazil, 2024. **Image source:** br.freepik.com

Below, the summaries of the main results of the selected studies are presented (figure 3).

The results pointed to sociocultural barriers regarding paternal participation in caring for a critically ill child, with this care being a role assigned to women. When her child becomes ill, the mother abandons her job, home, husband, other children, and her social life to dedicate herself to the critically ill child, while the father assumes a supporting role in this activity. There is a maternal tendency to believe that no one is capable of taking care of their child, that is, for the mother, only she is qualified for such an activity.

Regarding gender barriers, the sexist social stigmas in which men have been socialized suggest that the father is not qualified to care for his child. The need to return to work, the archaic conception of masculinity in which the father is the provider and the mother the caregiver, the lack of experience and ability to care for a critically ill child leads the maternal figure to be considered by parents as the person most capable of recognizing the illness and caring for the sick child. Furthermore, the prejudice against the male gender associated with the structure of collective wards makes it difficult for the father to remain with the child, since the joint presence of the father of one child with the mother of another in the same unit would be unfeasible, turning the father figure into a visitor and limiting his participation in the critically ill child care.

Regarding the family, psycho-emotional, and socioeconomic impact, it was observed that a critically ill child causes harm to family dynamics, resulting in poor quality in family functioning, division of parental functions, psychological and marital breakdown. For the father, the greatest difficulties related to caring for a critically ill child are financial, professional, social, emotional, as well as lack of time for self-care, high treatment costs, and lack of support from other family members. The experience of having a seriously ill child is permeated by psychological and emotional disorders, such as anxiety, depression, stress, fear, anguish, insecurity, and frustration.

Regarding the paternal form of care, studies have revealed that when a child becomes ill, direct paternal care for the child is limited, since the father believes he does not have the skills and knowledge to care for the child. Furthermore, the limited paternal participation in caring for a child with a serious illness is related to the man’s work activities. On the other hand, the father finds other ways to maintain the bond and participate in the care of the hospitalized and critically ill child, such as through skin-to-skin contact (kangaroo care), touch, caresses, hospital visits, talking to the child, and expressing positive feelings. In the home context, paternal care is related to seeking information about the child’s illness to promote positive changes in the child’s health condition, sleep and rest, food, hygiene, and leisure with moments of recreational interaction, such as playing.

Regarding the paternal role, it was found that the idea of paternity has been changing over time. Currently, men are more involved in caring for their children compared to their ancestors. As a way of being useful and participating in the care of the critically ill child, the father assumes important roles with the mother, with the child, and with the family unit. Regarding the mother, the father plays the role of emotional support, maternal support in coping with the child’s illness and comfort, strength and affection during the child’s hospitalization, providing moments of maternal rest and encouraging his partner to be absent from the hospital environment for a short period. With a child, the paternal role focuses on providing emotional support, being patient, dedicated, and demonstrating love and affection, making the child feel safe and easing their painful experiences. In addition to being the provider and head of the family, the father’s role within the family unit includes ensuring the well-being, protecting and supporting other family members, and taking on household chores, including caring for other children at home.

Regarding the benefits of paternal participation in caring for a critically ill child, the included studies showed that the father recognizes his importance in participating in the upbringing of his child, his role being fundamental for the child’s development. Thus, the father’s participation in sharing care for the critically ill child directly contributes to the quality of care provided by the health team, and is considered a beneficial and positive experience. A present father transmits love, comfort, security to the child, increasing intimacy with the child and having the opportunity to enhance his role.

## DISCUSSION

Throughout history, the role of men in caring for children has evolved considerably. At the beginning of the 20th century, fatherhood was considered a secondary duty to paid work. However, since the 1950s and 1960s, behavior towards parenthood has changed, encouraged, in most cases, by the progressive insertion of women into the job market. As a result, the father began to play an important role in raising children and, in many Western cultures, the father figure became actively involved in childcare. Even though social advances have occurred, inequality still exists in the role of men and women in the daily lives of families of critically ill children, with the mother taking on most of the caregiving duties^(64,65)^.

Martins and Reis^(66)^, in their studies, revealed that mothers consider themselves the main caregivers of their children, highlighting little paternal involvement. For them, even though the father is considered present in raising his children, when a child becomes ill, the man does not play any role in providing care for the illness. Furthermore, the mother attributes caring for the sick child as being hegemonically a maternal role.

Therefore, the sharing of parental care for the institutionalized child does not occur, since the mother remains with the child all the time, limiting paternal participation. This decision is supported by the mother’s perception that her children are more relaxed in her presence. Furthermore, the mother does not consider the father to be as capable of caring for the sick child, especially in situations of serious illness, as she is^(67)^.

For decades, fathers have been passive when it comes to caring for their children, with this role being culturally reserved for women who, from a young age, are already guided and nurtured towards the maternal universe, leaving men with little opportunity to develop paternity. Since the responsibility for caring is socially attributed to the role of women in society and is reproduced between generations, it becomes important to deconstruct social and identity representations about the retrograde model of father and reveal a new paternal figure^(68,69)^.

This process of continuous transformation and reconstruction, which is reflected in new concepts of family, has restructured what was called the provider father into what we want today: a fatherhood that is co-responsible for the reproduction and upbringing of his children^(70)^. This way, fatherhood is not limited to discipline and the provision of financial resources, but also participation in daily care from early childhood through school life^(71)^.

This paradigm shift requires the father figure to have knowledge about child care, so that the father can carry out his role with independence, safety and effectiveness, resulting in benefits in the biopsychosocial development of his offspring^(72)^.

However, the reality is controversial. Authors point out that the duty to return to work and the old idea of the man as the “breadwinner” and the mother as the caregiver, as well as the fear and lack of experience and paternal ability to care for a sick child, lead to mother being seen by the father as the most capable person to care for the sick child. Furthermore, the father figure feels responsible for providing for the family’s material needs and, as a result, is not directly involved in caring for the child, assuming a mother supporting role^(66,73)^.

It should be noted that there are legal weaknesses regarding the paternal role, in which workplaces do not recognize paternity and do not guarantee their rights assured in the various public policies that encourage fathers participation in the child’s development, such as the companion law, the paternity leave, and the Statute of the Child and Adolescent^(74,75)^.

Likewise, with regard to the lack of guarantees in the job market, there is labor informality, since many self-employed or informal workers do not have a stable financial life, forcing men, even when their children are ill, to maintain their work activities to cover their obligations as household heads. Thus, informality and long working hours challenge the exercise of paternity^(76)^.

The literature highlights other factors related to health services that hinder the promotion of active fatherhood, such as the feminization of childcare, paternal invisibility, short duration of paternity leave, idealization of a father supporting the mother, gender segregation in the parental role and characteristics of hospital institutions. Such adversities contribute to limited paternal participation in caring for the sick child and poor support for the father in health institutions^(74,77)^.

The hospital services scenario is a space where paternal incorporation is difficult, because in addition to not recognizing paternity, the physical infrastructure does not provide the welcome and comfort for their inclusion. The asymmetry in the recognition of father figures by the health team contributes to the father’s distancing. This strictly maternal-child representation of child care must be broken by health professionals, directing their attention to the family^(67,78,79)^.

In turn, health professionals recognize the importance of sharing care with the family and involving the father figure in caring for the child through the division of parental responsibilities. However, there is a lack of knowledge about the real meaning and ways to approach and apply family-centered care and the father figure during the hospitalization process of a critically ill child^(80,81)^.

In general, professionals were trained to direct guidance and attention to the primary caregiver, that is, the one who is present most of the time during the child’s hospitalization. When asked about who accompanies the child in the hospital environment, professionals say it is the maternal figure, with the father being absent at this time. For them, the primary caregiver for the sick child has already been pre-established by the family unit and generally the person who assumes this role is the person who does not work or who has given up work to dedicate themselves exclusively to the sick child, that is, the mother. Thus, paternal involvement in childcare is less expressive than that of mothers, which hinders the creation of a bond between the professional and the father, resulting in the health team not recognizing paternity^(82,83,84)^.

Studies show that women, as they are primarily responsible for caring for children with serious illnesses, face drastic changes in their routine and lifestyle, resulting in psycho-emotional imbalance. The large amount of time invested in caring for a sick child, combined with the absence of a father, causes mothers to experience loneliness and sleep deprivation, and to feel overwhelmed. The maternal figure tends to prioritize caring for the child at the expense of her own personal life, which is why they find themselves tired, depressed, stressed, and with a poor quality of life^(85,86,87,88)^.

As a result, one can infer how important father participation is in caring for the sick child, with his involvement being beneficial for maternal health, since the father being present provides psycho-emotional support for the mother and strengthens the father/mother and child trinomial in a healthy sharing of parental responsibilities, providing a healthy maternal experience in coping with the illness of the critically ill child^(89,90)^.

The benefits of paternal involvement in caring for a sick child transcend those related to the mother, also bringing improvements to the child’s general condition. The involved father promotes emotional well-being for his children, reducing stress and favoring the child’s cognitive development^(91,92)^.

The literature attributes advantages in the child’s biopsychosocial development related to the paternal presence. These include the consolidation of the bond and greater quality in the parent-child relationship, which favors the formation of the child’s identity; emotional support and promotion of child resilience in the face of adversity; and strengthening of emotional bonds, which results in a more secure child, with high self-esteem, empathy, and solidarity. Furthermore, the presence of the father figure contributes to the child’s treatment and recovery, resulting in a reduction in hospital stay time^(83,93,94)^.

When experiencing his child’s illness, the man redefines his paternal role, wishing not only to fulfill the role of family provider, but also to demonstrate interest in getting involved in every way in the child’s care. The new father takes on the role of caring for the mother, his sick child, the home, and the family^(95)^.

In what regards the maternal figure, the father plays his role by being attentive to the woman’s psycho-emotional needs, embracing her fears and insecurities, providing support and alleviating physical, mental and psychological exhaustion. In her most distressing and painful moments, the words of comfort and hope spoken by the fathers serve as strength and encouragement, helping the mother to face her child’s illness in a calm, confident, and optimistic way^(96,97)^.

Research on fatherhood confirms that the father figure has a positive impact on the development of a child’s social skills, as well as promoting psycho-socio-emotional well-being, helping with the child’s self-confidence, improving school performance, and reducing behavioral problems^(98,99,100)^.

When the child is sick, the father plays his role by protecting and ensuring the child’s development. It is up to the father to be affectionate, attentive, welcoming, demonstrate security, minimize the child’s suffering, and participate in the care of the institutionalized child^(101,102)^.

In the family context, the paternal role goes beyond the hospital environment, with the father being responsible for maintaining the integrity and functioning of the family unit. His functions are focused on supporting and welcoming the other members of the family, such as providing support and caring for the other children at home and carrying out household chores^(67,73,79)^.

Studies also point to a transition in the paternal role, revealing that fathers are increasingly concerned about participating in the care of children with serious illnesses, seeking to accompany their children to medical appointments and being present during the illness and treatment process. In this sense, the father figure is involved in the emotional and physical care of his children, performing various forms of care, among which the creation of an emotional bond, education, leisure, food, hygiene, diaper changing, sleep and rest and even specific care related to illness stand out^(64,95,103)^.

However, there are situations in which the father does not find time to get involved in the care or even believes he does not have the knowledge and skills to care for the sick child. In these cases, the mother hegemonicaly assumes the care for the sick child. The main factor in the lack of paternal time is related to the multiple roles that the father assumes, in which he needs to divide himself among paid work to provide for the family, care for a sick child, support for his partner, responsibility for the other children, and household chores. Thus, paternal participation in caring for a critically ill child is limited^(88,103,104)^.

To acquire the skills to exercise his paternal role and provide the care that his sick child needs, the father seeks information about the illness, treatment, and care directed at the child’s health on the internet, books, and television, which is a mechanism he has found to help his son. It is important to note that when a man creates means to meet his child’s psycho-emotional and physical needs, which includes health care, food, hygiene, protection, support and affection, he fulfills a role called fatherhood^(95,105,106)^.

The kangaroo method as a strategy to include the father in the care of the child hospitalized in neonatal therapy units has been widely discussed in the literature. The results show that the kangaroo care strengthens the emotional bond between the father-child dyad, favors greater paternal participation in the process of caring for the newborn, and redefines the condition of being a father, since it allows the man to experience fatherhood in a complete, meaningful way, and aware of his role as caregiver. The father, when experiencing these benefits, feels more confident in caring for the hospitalized newborn. This awakens the desire to stay with the child for longer^(107,108,109,110,111,112)^.

It should be highlighted that the neonatal intensive care unit is a challenging environment for parents. For them, having a child admitted to this health service means serious illness and risk of death. High levels of stress, suffering, and fear of losing the child are experienced by parents^(113)^.

Studies on the psychological shocks suffered by parents when faced with their child’s serious illness reveal painful and frightening experiences. Parents are affected by intense emotional imbalance that is expressed through suffering, sadness, anxiety, anguish, despair, hopelessness, guilt, fear of death, uncertainty, frustration and incapacity, which is a worrying, challenging, and exhausting condition for all family members^(114,115,116)^.

The father is also affected by his child’s illness, hospitalization and treatment, experiencing fear and terror at the possibility of his child’s death, frustration at having a sick child with limitations, despair and sadness at witnessing the child’s health condition, and uselessness at not being able to alleviate his child’s suffering. However, the father expresses his feelings differently from the mother. They express their pain and concerns through crying and appear irritated and nervous, but they try not to cry in front of their wife and family, they avoid talking about the problem, and adopt an introspective behavior, remaining more silent than usual^(51,67)^.

The diagnosis of a serious illness in a child results in changes in family dynamics and routine, adaptations of parental roles, and restrictions on the parents’ social and occupational lives. The illness and hospitalization of the child causes financial harm to the family unit due to the reduction in work activities and even the abandonment of employment, and in most cases, it is the woman who gives up her professional life to dedicate herself fully to the sick child. This reality is reflected in low quality of life and leisure for men, social isolation, excessive responsibilities, lack of time and priority for self-care and an overload of financial responsibilities for the father. Furthermore, there is a negative impact on interpersonal family and marital relationships^(117,118,119,120^.

Faced with family reorganization to meet the needs of the sick child, the family nucleus faces distancing in relationships and a lack of coexistence among its members. The illness of a child is seen by parents as a disturbing process, permeated by unhappiness and psycho-emotional exhaustion, resulting in the rupture of family and marital identity^(117,121,122)^.

A healthy married life requires physical and psychological balance so that an intimate and loving relationship can be established between the couple, which does not occur in parents of sick children, since the child’s illness is the main focus of attention in the parents’ lives. Therefore, the parents’ marital relationship is mediated by unhappiness, stress, and emotional distress. Moreover, a child’s illness causes physical and mental exhaustion, loss of privacy, sexual disinterest, conflicts, and impaired communication in parents, contributing to marital distress and separation^(123,124)^.

Considering the importance of highlighting paternity and encouraging the inclusion of fathers in the care of critically ill children, we believe we are making a significant contribution to the academic community, given that the training of health and nursing professionals does little to explore family-centered care and shows a lack of knowledge about the father figure. Consequently, the results of this review contribute to teaching, research, and the construction of an integrated curriculum that incorporates into its learning content subjects about the family unit and the paternal role with a view to qualified professional training.

This study also contributes to the sociocultural deconstruction of gender ideals regarding parental roles by revealing paternal suffering in cases where the man’s paternity is marginalized in relation to the life of his sick child, the factors that contribute to the father not actively participating in the care of a critically ill child, and the benefits that the father’s presence promotes in the health and development of the child, in maternal well-being, and in the care provided by nursing professionals.

As limitations of this scoping review, we highlight the selection of studies only in English, Spanish, and Portuguese, the inclusion of only the first ten pages of Google Scholar, and the unavailability of the full text of some articles, which may have hindered data collection and the selection of relevant works. Another limiting factor was the research context, since the topics of paternal care and gender equality are rarely addressed in the scientific literature, especially when associated with the care of a father for a critically ill child.

## CONCLUSION

The results of this review allowed mapping and describing paternal participation in caring for a critically ill child. Thus, this study found that father participation in caring for a critically ill child is limited. Even with the benefits of paternal involvement in caring for a sick child, the father faces obstacles to exercising his active paternity related to sociocultural and gender issues regarding parental roles. Furthermore, it was found that being the father of a critically ill child has a negative impact on a man’s life, causing economic, family, and psycho-emotional impacts.

Gaps identified in the academic training of health professionals on paternity and family-centered care are highlighted, showing the importance of training, even during undergraduate studies, to work in child health care, as many professionals are faced with historical, sociocultural, and gender stigmas related to parental roles, resulting in inhumane and fragmented care for the mother, father, and child triad and, as a consequence, limiting the exercise of paternity.

Finally, it is worth mentioning that new research must be carried out to investigate issues that have not yet been sufficiently explored and with the necessary methodological improvements to advance the understanding of the sociocultural and gender repercussions that influence and limit the exercise of paternity, the paternal role, and the benefits of the paternal presence and of father involvement in the care of a child with a serious illness.

In addition, future studies should aim to provide verticalized knowledge about paternity to health professionals, researchers, and public policy makers, with the aim of supporting the incorporation of strategies in care practice and in child and adolescent health services, improving the paternal experience when caring for a critically ill child, involving the paternal figure in family relationships, intensifying the humanization of care for the family unit, promoting actions that strengthen the father-child bond and reveal active paternity.
