# Supplementary material for: Father participation in the care of a critically ill child: a scoping review
Source: Rev Esc Enferm USP. 2025 Apr 28;59:e20240278. doi: 10.1590/1980-220X-REEUSP-2024-0278en (PMC12039821; doi:10.1590/1980-220X-REEUSP-2024-0278en)
Supplement: Translation

# Translation: A participação do pai no cuidado ao filho com doença grave: revisão de escopo

**DOI:** 10.1590/1980-220X-REEUSP-2024-0278pt

**Authors:** Rafaela Sterza da Silva, Cinara Bozolan Coppo, Edrian Maruyama Zani, Adriana Valongo Zani Zani

**Affiliations:** 1 Universidade Estadual de Londrina, Programa de Pós-graduação em Enfermagem, Londrina, PR, Brasil.

**Keywords:** Pai, Relações Pai-Filho, Comportamento Paterno, Cuidado da Criança, Doença Catastrófica

## Abstract

**Objetivo:**

Mapear e descrever os estudos existentes na literatura sobre a participação do pai no cuidado ao filho com doença grave.

**Método:**

Revisão de escopo segundo a metodologia do JBI. A busca aconteceu em outubro de 2023, com estudos publicados na íntegra em português, inglês ou espanhol, sem limitação temporal e indexados em bases de dados e portais de teses e dissertações. Dois revisores realizaram a leitura do título e resumo dos estudos e exportaram os dados para uma planilha do *Microsoft Excel®* para agrupamento e remoção das duplicidades. A síntese dos dados deu-se de forma escrita e visual, mediante diagramas.

**Resultados:**

A busca resultou em 961 estudos, sendo elegíveis 38 estudos para compor a amostra. A análise dos estudos permitiu identificar que a participação paterna no cuidado ao filho com doença grave envolveu os benefícios para o filho, para a mãe e para a família; as formas de cuidar paterna; o papel social do pai; os impactos psicoemocionais, socioeconômicos e familiares e as barreiras de gênero e socioculturais acerca da figura paterna.

**Conclusão:**

A participação paterna no cuidado ao filho com doença grave revelou-se um fenômeno complexo que requer a desconstrução de estigmas históricos, socioculturais e de gênero relacionados aos papéis parentais.

## INTRODUÇÃO

Por influência política, econômica, social e religiosa, a unidade familiar passou por grandes transformações com o passar dos anos no que se refere ao seu conceito, estrutura, dinâmica e papel desempenhado por cada integrante. A família é entendida como base da organização de uma sociedade e, por séculos, foi constituída alicerçada no patriarcado, onde mulheres não tinham voz e a elas cabia apenas o papel de cuidar da casa e criar os filhos, enquanto ao homem competia a função de autoridade e provedor familiar^(1)^.

O papel do pai na sociedade contemporânea evoluiu significativamente, ultrapassando a função de mero provedor financeiro e autoridade da família. Hoje, os pais estão cada vez mais envolvidos no cuidado e na educação dos filhos, desempenhando um papel ativo na transmissão de valores, afeto e proteção^(2)^. Tal transformação na paternidade reflete na ressignificação do conceito de pai, que agora se vê como protagonista na criação dos filhos, buscando estabelecer vínculos afetivos profundos e participar ativamente do cotidiano familiar^(3,4)^.

No entanto, a sociedade ainda carrega padrões tradicionais que frequentemente relegam o pai a um papel secundário na criação dos filhos, especialmente em contextos de cuidado. A figura paterna, apesar de seu desejo de maior envolvimento, enfrenta obstáculos culturais e sociais que associam a parentalidade principalmente à figura materna^(5,6)^. Essa visão tradicional limita o envolvimento paterno, fazendo com que muitos pais se sintam coadjuvantes no processo de criação e cuidados com os filhos^(7)^.

Tal dualidade de papéis é exacerbada em situações de adoecimento infantil, onde a figura paterna tende a retornar a sua função de provedor familiar, refletindo no conflito entre a concepção moderna de paternidade e a ideologia retrógrada de masculinidade. Nesse contexto, muitos pais ainda se sentem pressionados a assumir as responsabilidades financeiras da família, enquanto o cuidado direto ao filho doente está centrado nas mães, perpetuando os estigmas de gênero enraizados na sociedade^(8,9)^.

A literatura científica destaca a importância do envolvimento paterno no cuidado à criança, especialmente em casos de doença grave. Estudos apontam que a participação ativa do pai no cuidado com o filho enfermo pode impactar positivamente no desenvolvimento da criança e melhorar a qualidade da assistência prestada^(10,11)^. Contudo, a participação ativa do pai não é um consenso entre os estudiosos, evidenciando que muitos pais ainda operam sob concepções tradicionais de gênero, limitando o exercício da paternidade^(12)^.

Embora o envolvimento do pai no cuidado ao filho doente seja controverso, a literatura também não oferece uma definição precisa de doença grave na infância. No entanto, ela estabelece uma lista de enfermidades caracterizadas por seu desenvolvimento progressivo e duradouro, com potencial para causar incapacidades, dependência de cuidados e ameaça de morte, ressaltando a complexidade das condições que exigem cuidados contínuos e intensivos^(13)^. No contexto deste estudo, doença grave foi definida como uma condição clínica com potencial para causar sérias consequências à saúde, podendo levar à incapacidade significativa, complicações severas ou até mesmo à morte. Normalmente, essas doenças requerem hospitalizações prolongadas devido à necessidade de inúmeras intervenções médicas.

Durante o tratamento e a reabilitação dessas crianças, a participação paterna frequentemente se mostra mínima ou até ausente, evidenciando a necessidade urgente de maior conscientização e apoio para promover a equidade no envolvimento parental frente ao filho com doença grave^(14)^.

Estudos demonstram avanços no conhecimento acerca da paternagem, influência sociocultural e de gênero no exercício da paternidade, papel paterno na relação pai e filho e a função paterna no desenvolvimento infantil, evidenciando recente interesse por parte dos pesquisadores sobre a figura paterna^(15,16,17,18,19,20,21)^. Contudo, pouco se sabe sobre o exercício da paternidade frente a um filho gravemente enfermo. Em uma pesquisa na *Open Science Framework e* na *International prospective register of systematic reviews* (PROSPERO), não se identificaram revisões de escopo e revisões sistemáticas existentes que respondam à pergunta: como ocorre a participação do pai no cuidado ao filho com doença grave?

Em decorrência do cenário apresentado, com intuito de revelar a figura paterna e contribuir para a consolidação do conhecimento acerca do cuidado paterno ao filho com doença grave, o objetivo dessa pesquisa foi mapear e descrever os estudos existentes na literatura sobre a participação do pai no cuidado ao filho com doença grave.

## MÉTODO

### Desenho do Estudo

Trata-se de revisão de escopo, que buscou explorar, mapear, sintetizar evidências científicas e identificar as lacunas de conhecimento acerca da presença e participação do pai no cuidado ao filho com doença grave. O presente estudo foi desenvolvido de acordo com o manual JBI^(22)^ e as diretrizes do *Preferred Reporting Items for Systematic Reviews and Meta-Analyses extension for Scoping Reviews* (PRISMA-ScR) *Checklist and Explanation* para a transparência na redação do relatório da revisão^(23)^.

O protocolo dessa revisão foi cadastrado no *Open Science Framework* (OSF), sob a identificação https://osf.io/5fzrg/DOI 10.17605/OSF.IO/5FZRG.

Para elaboração desta revisão, foram seguidas as cinco etapas^(24)^: 1) identificação da questão de pesquisa; 2) identificação dos estudos relevantes; 3) seleção e avaliação inicial dos estudos; 4) análise dos dados; e 5) agrupamento, síntese e apresentação dos dados.

### Coleta de Dados

Para a formulação da questão de pesquisa (etapa 1), utilizou-se o mnemônico PCC (População, Conceito e Contexto), descrito no quadro 1.

**Quadro 1:** Estratégia PCC (População, Conceito e Contexto) – Londrina, PR, Brasil, 2024.

	PCC	Descrição
P	População	Pai
C	Conceito	Estudos que abordem o cuidado paterno
C	Contexto	Cuidado da criança, Doença grave ou catastrófica

Fundamentado nessa estratégia, elaborou-se a pergunta de pesquisa: Como ocorre a participação do pai no cuidado ao filho com doença grave?

A identificação dos estudos relevantes (etapa 2) envolveu a seleção das bases de dados, definição das estratégias de busca a partir dos descritores e operadores booleanos, e a formulação dos critérios de inclusão e exclusão. A busca deu-se nas seguintes bases de dados: CINAHL (*Cumulative Index to Nursing and Allied Health Literature*), Embase (*Excerpta Medica dataBASE*), *Scopus*, *Web of Science*, SciELO (*Scientific Electronic Library Online*), PubMed e a Biblioteca Virtual em Saúde (BVS). Para investigação da literatura cinzenta, utilizou-se o *Google Scholar* e o Catálogo de Teses e Dissertações da Coordenação de Aperfeiçoamento de Pessoal de Nível Superior (CAPES). A recuperação dos estudos no *Google Scholar* foi realizada nas 30 (trinta) primeiras páginas. Ressalta-se que as estratégias de busca foram ajustadas em concordância com as particularidades de cada base de dados.

A coleta de dados ocorreu em outubro de 2023 por meio do portal de periódicos da Coordenação de Aperfeiçoamento de Pessoal de Nível Superior (CAPES), via Comunidade Acadêmica Federada (CAFe). Foi empregado o recurso “busca avançada” a partir dos descritores MeSH (*Medical Subject Headings*) e DeCS (Descritores em Ciências da Saúde) e operadores booleanos *AND* e *OR*. No Catálogo de Teses e Dissertações da CAPES foi aplicada a estratégia de busca do termo solitário e o cruzamento dos termos sem os operadores booleanos. A estratégia compreendeu o cruzamento dos termos entre si, conforme mostrado a seguir:

- Descritores em Ciências da Saúde (DeCS): Pai *AND* Relações Pai-Filho *OR* Comportamento Paterno *AND* Cuidado da Criança *AND* Doença Grave.
- *Medical Subject Headings* (MeSH): *Fathers AND Father-Child Relations OR Paternal Behavior AND Child Care AND Critical Illness.*

Foram incorporados os descritores não controlados “filho doente” e “cuidado do pai”. A seguir, no quadro 2, está detalhada a estratégia de busca empregada em cada base de dados.

**Quadro 2:** Elaboração da estratégia de busca nas diversas bases de dados/portais – Londrina, PR, Brasil, 2024.

Base de Dados	Estratégia de busca
BVS	#1 Pai *AND* Relações Pai-Filho *OR* Comportamento Paterno *AND* Cuidado da Criança *AND* Doença Grave; #2 Pai *AND* Cuidado da Criança *AND* Doença Grave; #3 Pai *AND* Cuidado da Criança *AND* “Filho doente”; #4 Comportamento Paterno *AND* Filho Doente *AND* Doença Grave
CINAHL	#1 *Fathers AND Father-Child Relations OR Paternal Behavior AND Child Care AND Critical Illness*; #2 *Fathers AND Child Care AND Critical Illness*
PubMed	#1 *Fathers AND Father-Child Relations AND Child Care AND Critical Illness*; #2 *Fathers AND Child Care AND Critical Illness*; #3 *Paternal Behavior AND Child Care AND Critical Illness*; #4 *Paternal Behavior AND “Sick Child” AND Critical Illness*; #5 *Paternal Behavior AND “Sick Child”*
Embase	#1 *Father AND Father child relation OR Paternal Behavior AND Child Care AND Critical Illness*; #2 *Father AND Child Care AND Critical Illness*; #3 *Father child relation OR Paternal Behavior AND Child Care AND “Sick Child”*; #4 *Father child relation OR Paternal Behavior AND “Sick Child”*; #5 *Father AND Child Care*
Scopus	#1 *Fathers AND Father-Child Relations AND Child Care AND Critical Illness*; #2 *Father AND Child Care AND Critical Illness*; #3 *Paternal Behavior AND “Sick Child”*
*Web of Science*	#1 *Fathers AND Father-Child Relations OR Paternal Behavior AND Child Care AND Critical Illness*; #2 *Fathers AND Child Care AND Critical Illness*; #3 *Paternal Behavior AND “Sick Child”*; #4 *Fathers AND Child Care AND “Sick Child”*
*SciELO*	#1 *Fathers AND Father-child relation OR Paternal Behavior AND Child Care AND Critical Illness*; #2 *Fathers AND Child Care AND Critical Illness*; #3 *Father-child relation AND “Sick Child*”; #4 *Fathers AND “Sick Child” AND Critical Illness*; #5 *Fathers AND Child Care*; #6 *Fathers AND “Sick Child”*
Google acadêmico	#1 Pai *AND* Relações Pai-Filho *OR* Comportamento Paterno *AND* Cuidado da Criança *AND* Doença Grave
CAPES	#1 “Cuidado do Pai”; #2 “Filho Doente”; #3 Relações Pai-Filho; #4 Comportamento Paterno

### Critérios de Seleção

Os critérios de elegibilidade dos estudos foram estabelecidos de forma a assegurar que somente documentos pertinentes ao escopo da revisão sejam incluídos na análise, garantindo a obtenção de uma revisão de alta qualidade, sustentada em evidências relevantes sobre a presença e participação do pai no cuidado ao filho com doença grave.

Fizeram parte dessa revisão estudos publicados nas bases de dados e literatura cinza estabelecidas que envolveram o pai como população e abordaram o conceito do comportamento paterno, relação pai-filho e a participação paterna no cuidado ao filho frente ao contexto da doença grave ou catastrófica.

Desse modo, como critérios de elegibilidade, estabeleceram-se: publicações sem recorte temporal, nos idiomas português, inglês e espanhol, de qualquer desenho metodológico, disponíveis na íntegra e gratuitamente em meio eletrônico, como também dissertações e teses, que responderam à pergunta de pesquisa. Excluíram-se estudos do tipo editorial, carta-resposta, artigos de opinião, e os documentos duplicados foram computados apenas uma vez.

### Extração e Análise dos Dados

A seleção e avaliação inicial dos estudos (etapa 3) envolveram a participação de dois revisores de forma independente que, preliminarmente, realizaram uma leitura acurada do título e resumo dos estudos encontrados. Em seguida, os dados dos estudos elegíveis foram exportados para uma planilha do *Microsoft Excel®* para que transcorresse o agrupamento e remoção das duplicidades. Para concluir, os revisores realizaram a leitura dos materiais na íntegra, aplicando-se mais uma vez os critérios de elegibilidade para posterior extração dos dados.

Foi empregada a investigação das citações de todos os estudos que atenderam os critérios de inclusão, com o objetivo de identificar estudos extras. Os estudos elegidos nessa nova investigação foram analisados igualmente aos demais, conforme supracitado, e identificados separadamente na apresentação dos resultados.

As divergências entre os pesquisadores durante o processo de seleção dos estudos foram resolvidas por um terceiro revisor, com expertise na temática, que forneceu o parecer final. Os resultados quantitativos de cada base, estudos excluídos e seus motivos, total de estudos incluídos para análise dos dados e síntese, foram descritos por meio de um diagrama de fluxo.

A análise dos dados (etapa 4) compreendeu a análise e categorização dos dados extraídos com o intuito de identificar padrões, tendências e lacunas na literatura. Os estudos foram analisados por dois autores independentes e um quadro sinótico, elaborado pelos revisores, serviu para sistematizar os dados extraídos, o qual compunha-se das seguintes informações: identificação do estudo, nome dos autores, ano de publicação, título, tipo de publicação, país de origem, periódico, tipo de estudo, objetivo do estudo e principais resultados e considerações.

Ressalta-se que o quadro sinótico para recolha dos dados foi previamente avaliado pelos autores por meio de teste piloto. O teste piloto para extração dos dados foi empregado por dois pesquisadores, que utilizaram a mesma estratégia de busca supramencionada e testaram o quadro sinótico em 10 estudos de forma aleatória para alinhar os critérios de elegibilidade e assegurar que todos os dados relevantes fossem comtemplados. Após a busca piloto nas bases de dados e teste realizado no quadro sinótico, os dois pesquisadores responsáveis pelo teste piloto reuniram-se com os demais autores para discussão e avaliação da aplicabilidade do quadro sinótico, da estratégia de busca e se a metodologia empregada possibilitava o alcance dos objetivos da pesquisa. Destaca-se que, após discussão entre os pesquisadores, não houve necessidade de reformulação do processo de busca e extração dos dados.

O agrupamento, síntese e apresentação dos dados (etapa 5) ocorreram de forma escrita e visual, mediante diagramas, com propósito de conceber visualidade e compreensão dos resultados. Em consonância, realizou-se uma análise crítica dos resultados dissertada de modo descritivo e reflexivo das evidências obtidas.

### Aspectos Éticos

Realizou-se a pesquisa segundo os princípios éticos e as diretrizes metodológicas cabíveis, cumprindo-se as boas práticas em pesquisa científica e respeitando-se os direitos autorais dos estudos citados. Por se tratar de um estudo de revisão baseado na análise de dados secundários disponíveis publicamente, e por não envolver diretamente a participação de seres humanos, não houve questões éticas que exigissem a apreciação de um comitê de ética.

## RESULTADOS

A busca resultou em 961 estudos, sendo 135 publicações removidas por duplicidade. Assim, 826 estudos foram analisados a partir do título e resumo, tendo 694 documentos excluídos por não atenderem a temática, pela indisponibilidade de texto completo e por se tratarem de estudos de revisão, restando 132 documentos para avaliação na íntegra quanto aos critérios de elegibilidade. Desses, foram excluídos 96 estudos resultando na seleção de 36 documentos. Ressalta-se que duas publicações foram adicionadas a partir da lista de referências, totalizando 38 estudos para compor a amostra final.

A figura 1 ilustra o diagrama de fluxo do processo de seleção dos estudos conforme recomendações do JBI, adaptado do *Preferred Reporting Items for Systematic reviews and Meta-Analyses extension for Scoping Reviews Checklist* (PRISMA-ScR)^(25)^.

**Figura 1:** Fluxograma de seleção dos estudos adaptado do PRISMA-ScR^(25)^ – Londrina, PR, Brasil, 2024.

Os 38 estudos selecionados para compor os resultados dessa pesquisa foram categorizados e organizados em uma sequência linear de tempo crescente, de acordo com o ano de publicação, tipo de publicação, país de origem e base de dados (Figura 2). Ressalta-se que as citações da figura 2 foram enumeradas sequencialmente conforme a ordem das bases de dados utilizadas na estratégia de busca, sendo BVS^(26,27)^, CINAHL^(28,29,30,31)^, Embase^(32,33,34,35)^, PubMed^(36)^, SciELO^(37,38,39,40,41)^, Scopus^(42)^, *Web of Science* ^(43,44)^, CAPES^(45)^, *Google Scholar* ^(46,47,48,49,50,51,52,53,54,55,56,57,58,59,60,61)^ e pesquisa de citações/outras fontes^(62,63)^.

**Figura 2:** Linha do tempo dos estudos selecionados segundo ano de publicação, tipo de publicação, quantidade de publicação de acordo com o país de origem e porcentagem de publicação de acordo com o ano – Londrina, PR, Brasil, 2024.

Os anos de maior produção científica sobre a temática foram 2013^(36,41,50,56,59)^ e 2020^(34,35,43,54,55)^, totalizando cinco estudos cada um deles, seguidos pelos anos de 2019^(32,51,52,60)^ (n = 4); 2010^(30,31,53)^, 2012^(29,42,49)^ e 2018^(26,48,58)^ com três publicações cada; 2014^(40,45)^, 2016^(28,47)^, 2017^(27,38)^ e 2022^(37,61)^ com dois estudos em cada ano. Os anos com apenas uma publicação foram 1991^(33)^, 2004^(46)^, 2006^(57)^, 2008^(62)^, 2009^(44)^, 2015^(63)^ e 2021^(39)^.

Majoritariamente, o tipo de publicação das produções foram artigos científicos^(26,27,28,29,30,31,32,33,34,35,36,37,38,39,40,41,42,43,44,46,47,48,49,50,51,52,58,62,63)^ (n = 29). As dissertações^(45,53,54,55,56,57)^ representaram 15,7% dos estudos e tem-se apenas uma produção do tipo trabalho de conclusão de curso^(60)^ (TCC), monografia^(59)^ e tese^(61)^.

Os países de origem dos estudos foram variados, sendo o Brasil^(26,27,28,29,30,31,38,39,40,41,42,46,47,48,50,51,52,53,54,55,56,57,58,59,60,61,62,63)^ o país que mais desenvolveu estudos sobre o tema (n = 28), seguido pela Irlanda do Norte^(44,49)^ (n = 2), Colômbia^(37,45)^ (n = 2), Inglaterra^(33)^ (n = 1), Suíça^(34)^ (n = 1), Etiópia^(35)^ (n = 1), México^(43)^ (n = 1), Mali^(36)^ (n = 1) e Polônia^(32)^ (n = 1).

A análise dos estudos permitiu identificar que a participação paterna no cuidado ao filho com doença grave envolve três categorias: 1. Participação do pai no cuidado ao filho com doença grave, que discorre sobre os benefícios da participação paterna, as formas de cuidar e o papel paterno com o filho doente, com a unidade familiar e com a mãe; 2. Repercussões de ter um filho doente, na qual elucida os impactos psicoemocionais, socioeconômicos e familiares vividos pelo pai; 3. Obstáculos para o cuidar paterno, que retrata as barreiras de gênero e socioculturais acerca da figura paterna.

A seguir, são apresentadas as sínteses dos principais resultados dos estudos selecionados (figura 3).

**Figura 3:** Mapa mental dos principais resultados dos estudos incluídos na revisão de escopo – Londrina, PR, Brasil, 2024. **Fonte da imagem:** br.freepik.com

Os resultados apontaram para barreiras socioculturais acerca da participação paterna no cuidado ao filho com doença grave, sendo o cuidado à criança gravemente enferma um papel designado à mulher. Diante do adoecimento do filho, a mãe abandona seu trabalho, casa, marido, outros filhos e sua vida social para se dedicar ao filho com doença grave, enquanto o pai assume o papel de coadjuvante nessa atividade. Há uma tendência materna em acreditar que ninguém é capaz de cuidar do seu filho, ou seja, para a mãe, somente ela é qualificada para tal atividade.

No que tange as barreiras de gênero, os estigmas sociais machistas no qual o homem foi socializado sugere que o pai não é qualificado para cuidar de seu filho. A necessidade de retorno ao trabalho, a concepção arcaica de masculinidade em que o pai é provedor e a mãe cuidadora, a falta de experiência e habilidade em realizar os cuidados com o filho com doença grave faz com que a figura materna seja considerada pelos pais como sendo a pessoa mais capaz de reconhecer a doença e cuidar do filho enfermo. Ademais, o preconceito acerca do gênero masculino associado à estrutura das enfermarias coletivas dificulta a permanência paterna junto à criança, já que a presença conjunta do pai de uma criança com a mãe de outra na mesma unidade seria inviável, tornando a figura paterna um visitante e limitando sua participação no cuidado ao filho doente.

Acerca do impacto familiar, psicoemocional e socioeconômico, observou-se que uma criança gravemente enferma provoca prejuízos na dinâmica familiar, repercutindo em má qualidade no funcionamento da família, divisão de funções parentais, desarranjo psicológico e matrimonial. Para o pai, as maiores dificuldades relacionadas ao cuidado do filho com doença grave são de ordem financeira, profissional, social, afetiva, falta de tempo para o autocuidado, altos custos com o tratamento e falta de apoio de outros membros da família. A experiência de ter um filho gravemente doente é permeada por distúrbios psíquicos e emocionais, como ansiedade, depressão, estresse, medo, angústia, insegurança e frustação.

No que se refere à forma de cuidar paterna, os estudos revelaram que diante do adoecimento do filho o cuidado paterno direto com a criança é limitado, uma vez que o pai julga não possuir habilidades e conhecimentos para cuidar da criança. Além disso, a limitada participação paterna no cuidado ao filho com doença grave relaciona-se com as atividades de trabalho do homem. Por outro lado, o pai encontra outras formas de manter o vínculo e participar do cuidado com o filho hospitalizado e gravemente enfermo, como através do contato pele a pele (posição canguru), toque, carícias, visita hospitalar, conversar com o filho e expressar sentimentos positivos. No contexto domiciliar, o cuidado paterno relaciona-se com a busca de informações acerca da doença do filho para promover mudanças positivas na condição de saúde da criança, sono e repouso, alimentação, higiene e lazer com momentos de interação lúdica, como por exemplo o brincar.

No que concerne o papel paterno, constatou-se que a ideia de paternidade vem se modificando ao longo do tempo. Os homens atuais estão mais envolvidos no cuidado com seus filhos quando comparado com seus ancestrais. Como forma de ser útil e participar do cuidado com o filho gravemente doente, o pai assume papéis importantes com a mãe, com o filho e com a unidade familiar. Acerca da mãe, o pai desempenha a função de suporte emocional, apoio materno no enfrentamento da doença do filho e conforto, força e afago diante da hospitalização da criança, oferecendo momentos de descanso materno e encorajando sua companheira a se ausentar por curto período do ambiente hospitalar. Com o filho, o papel paterno concentra-se em promover apoio emocional, ser paciente, dedicado e demonstrar amor e afeto, fazendo com que a criança se sinta segura e amenizando suas experiências dolorosas. Além de ser provedor e chefe de família, o papel paterno com a unidade familiar abrange garantir o bem-estar, proteger e apoiar os demais membros da família e assumir os afazeres domésticos, incluindo cuidar dos outros filhos em casa.

Com relação aos benefícios da participação paterna no cuidado ao filho com doença grave, os estudos incluídos evidenciaram que o pai reconhece a sua importância na participação da criação do seu filho, sendo o seu papel fundamental para o desenvolvimento da criança. Deste modo, a participação do pai no compartilhamento do cuidado à criança gravemente enferma contribui diretamente na qualidade da assistência prestada pela equipe de saúde, sendo considerada uma experiência benéfica e positiva. O pai presente transmite amor, aconchego, segurança para a criança e aumenta a intimidade com o filho, tendo oportunidade de fortalecer o seu papel.

## DISCUSSÃO

Ao longo da história, o papel dos homens no cuidado das crianças evoluiu consideravelmente. No início do século XX, a paternidade era considerada um dever secundário em relação ao trabalho remunerado. Contudo, desde as décadas de 50 e 60 o comportamento em relação à parentalidade transfigurou, incitado, em grande parte dos casos, pela inserção progressiva da mulher no mercado de trabalho. Em razão disso, o pai passou a configurar um importante papel na criação dos filhos e, em muitas culturas ocidentais, a figura paterna envolveu-se ativamente no cuidado infantil. Mesmo que avanços sociais tenham ocorrido, a desigualdade ainda existe no nível do papel dos homens e das mulheres na vida cotidiana das famílias de crianças com doenças graves, cabendo à mãe a maior parte das funções de cuidadora^(64,65)^.

Martins e Reis^(66)^, em seus estudos, revelaram que as mães se consideram as principais cuidadoras de seus filhos, destacando pouco envolvimento paterno. Para elas, por mais que o pai seja considerado presente na criação dos filhos, diante do adoecimento da criança, no que se refere ao cuidado relativo à doença, o homem não exerce nenhuma função. Além do mais, a mãe atribui o cuidado com o filho doente como sendo hegemonicamente um papel materno.

Desse modo, o compartilhamento do cuidado parental da criança institucionalizada não acontece, já que a mãe permanece todo o tempo junto do filho, limitando a participação paterna. Essa decisão é sustentada na percepção materna de que os filhos, na sua presença, demonstram maior tranquilidade. Ademais, a mãe não considera o pai apto para cuidar da criança doente, principalmente em situações de doença grave, tão bem quanto ela^(67)^.

Durante décadas, o pai tem sido passivo no que diz respeito aos cuidados com o filho, sendo essa função culturalmente reservada às mulheres que, desde tenra idade, já são orientadas e nutridas para o universo maternal, sobrando ao homem pouca oportunidade de desenvolver a paternidade. Uma vez que a responsabilidade do cuidar é socialmente imputada ao papel da mulher na sociedade e se reproduz entre gerações, surge a importância de descontruir as representações sociais e identitárias acerca do retrógrado modelo de pai e revelar uma nova figura paterna^(68,69)^.

Esse processo de contínua transformação e reconstrução, que reflete em novos conceitos de família, reestruturou o que se chamava de pai do provedor no que hoje queremos: uma paternidade corresponsável pela reprodução e criação de seus filhos^(70)^. Dessa forma, a paternidade não se limita à disciplina e à disponibilização de recursos financeiros, mas também à participação nos cuidados diários desde a primeira infância até a vida escolar^(71)^.

Essa mudança de paradigma exige da figura paterna conhecimento sobre o cuidado infantil, para que o pai desenvolva seu papel com independência, segurança e eficácia, resultando em benefícios no desenvolvimento biopsicossocial de sua prole^(72)^.

No entanto, a realidade mostra-se controversa. Autores apontam que o dever de voltar ao trabalho e a velha ideia do homem como “ganha-pão” e da mãe cuidadora, assim como o medo e a falta de experiência e capacidade paterna para cuidar do filho enfermo, fazem com que a mãe seja tida pelo pai como a mais capaz para cuidar da criança doente. Além do mais, a figura paterna sente-se responsável por prover as necessidades materiais da família e, em razão disso, não se envolve diretamente no cuidado com o filho, assumindo um papel de coadjuvante materno^(66,73)^.

Ressalta-se a existência de fragilidades legais acerca do papel paterno, nas quais os locais de trabalho não reconhecem a paternidade e não garantem seus direitos assegurados nas diversas políticas públicas que incentivam a participação do pai no desenvolvimento do filho, como por exemplo a lei do acompanhante, licença paternidade e Estatuto da criança e do adolescente^(74,75)^.

Do mesmo modo, no que se refere à falta de garantias no mercado de trabalho, tem-se a informalidade laboral, já que muitos trabalhadores autônomos ou informais não possuem vida financeira estável, fazendo o homem, mesmo diante do adoecimento do filho, manter suas atividades de trabalho para custear suas obrigações como chefe de família. Assim, a informalidade e a longa jornada de trabalho desafiam o exercício da paternidade^(76)^.

A literatura evidencia outros fatores dificultadores da promoção da paternidade ativa relacionados com os serviços de saúde, como a feminização do cuidado infantil, invisibilização paterna, curta duração da licença paternidade, idealização de um pai coadjuvante da mãe, segregação de gênero no papel parental e características das instituições hospitalares. Tais adversidades contribuem para a limitada participação paterna no cuidado ao filho doente e pouco acolhimento do pai nas instituições de saúde^(74,77)^.

O cenário dos serviços hospitalares é um espaço de difícil incorporação paterna, pois além de não reconhecerem a paternagem, a infraestrutura física não propicia o acolhimento e o conforto para a sua inclusão. A dissimetria no reconhecimento das figuras parentais pela equipe de saúde colabora para o distanciamento do pai. Tal representação estritamente materno-infantil acerca dos cuidados com as crianças deve ser rompida pelos profissionais de saúde, direcionando o olhar para a família^(67,78,79)^.

Por sua vez, os profissionais de saúde admitem a importância de compartilhar o cuidado com a família e de envolver a figura paterna na assistência com a criança por meio da divisão das responsabilidades parentais. Contudo, falta conhecimento sobre o real significado e maneiras de abordar e aplicar o cuidado centrado na família e na figura paterna durante o processo de hospitalização da criança com doença grave^(80,81)^.

De modo geral, os profissionais foram formados para direcionar as orientações e a atenção para o cuidador principal, ou seja, aquele que se faz presente em maior parte do tempo durante a internação da criança. Quando questionados sobre quem acompanha o filho no ambiente hospitalar, os profissionais afirmam ser a figura materna, sendo o pai ausente nesse momento. Para eles, o cuidador principal da criança doente já foi preestabelecido pela unidade familiar e geralmente quem assume essa função é a pessoa que não trabalha ou que abdicou do trabalho para se dedicar exclusivamente ao filho enfermo, isto é, a mãe. Assim, o envolvimento paterno no cuidado infantil é menos expressivo do que o das mães, o que dificulta a criação de vínculo entre o profissional e pai, ocasionando no não reconhecimento da paternagem pela equipe de saúde^(82,83,84)^.

Estudos apontam que a mulher, por ser a principal responsável por cuidar das crianças com doenças graves, enfrenta drásticas mudanças em sua rotina e estilo de vida, resultando em desequilíbrio psicoemocional. O grande tempo investido no cuidado ao filho doente somado à ausência paterna faz com que as mães vivenciem a solidão, privação do sono e sintam-se sobrecarregadas. A figura materna tende a priorizar o cuidado com a criança à custa de sua própria vida pessoal, por isso encontram-se cansadas, deprimidas, estressadas e com má qualidade de vida^(85,86,87,88)^.

Em vista disso, infere-se o quão importante é a participação do pai no cuidado ao filho doente, sendo seu envolvimento benéfico para a saúde materna, uma vez que o pai presente serve de suporte psicoemocional para a mãe e fortalece o trinômio pai/mãe e filho em um saudável compartilhamento de responsabilidades parentais, proporcionando uma experiência materna salubre no enfrentamento da enfermidade da criança com doença grave^(89,90)^.

Os benefícios do envolvimento paterno nos cuidados ao filho doente transcendem aqueles relacionados à mãe, trazendo também melhorias no estado geral da criança. O pai participativo promove bem-estar emocional para seus filhos, reduzindo o estresse e favorecendo o desenvolvimento cognitivo da criança^(91,92)^.

A literatura atribui vantagens no desenvolvimento biopsicossocial da criança relacionadas à presença paterna. Dentre elas, tem-se a consolidação do vínculo e maior qualidade na relação pai-filho, o que favorece a formação da identidade do filho; suporte emocional e promoção da resiliência infantil diante das adversidades e fortalecimento de laços afetivos, o que repercute em uma criança mais segura, de autoestima elevada, empática e solidária. Além disso, a presença da figura paterna contribui para o tratamento e recuperação da criança, refletindo na redução do tempo de hospitalização^(83,93,94)^.

Ao vivenciar o adoecimento do filho, o homem ressignifica seu papel paterno, desejando não apenas desempenhar a função de provedor da família, como também demonstrar interesse em envolver-se de todas as maneiras no cuidado à criança. O novo pai assume o papel de cuidar da mãe, do filho doente, da casa e da família^(95)^.

No que se refere à figura materna, o pai exerce seu papel estando atento às necessidades psicoemocionais da mulher, acolhendo seus medos e inseguranças, promovendo apoio e amenizando o esgotamento físico, mental e psicológico. Em seus momentos mais angustiantes e dolorosos, as palavras de conforto e esperança dita pelos pais servem de força e ânimo, ajudando a mãe enfrentar o adoecimento do filho de maneira serena, confiante e otimista^(96,97)^.

Pesquisas sobre paternagem confirmam que a figura paterna impacta positivamente na formação das habilidades sociais do filho, bem como promove o bem-estar psico socioemocional, ajudando na autoconfiança da criança, melhorando o rendimento escolar e reduzindo problemas de comportamento^(98,99,100)^.

Com o filho doente, o pai exerce seu papel protegendo e garantindo o desenvolvimento da criança. Cabe ao pai ser afetuoso, atento, acolhedor, demonstrar segurança, minimizar o sofrimento da criança e participar dos cuidados com o filho institucionalizado^(101,102)^.

No contexto familiar, o papel paterno vai além do ambiente hospitalar, cabendo ao pai manter a integridade e funcionamento da unidade familiar. Suas funções estão voltadas para o sustento e acolhimento dos demais integrantes da família, como dar suporte e cuidar dos outros filhos em casa e realizar as tarefas domésticas^(67,73,79)^.

Estudos também apontam para uma transição da função paterna, revelando que cada vez mais o pai se preocupa em participar do cuidado da criança com doença grave, busca acompanhar o filho nas consultas médicas e estar presente durante no processo de adoecimento e tratamento. Nesse sentido, a figura paterna envolve-se no cuidado emocional e físico dos filhos, desempenhando várias formas de cuidar, dentre elas destacando-se a criação de vínculo afetivo, educação, lazer, alimentação, higiene, troca de fraldas, sono e repouso e até mesmo cuidados específicos relacionados à doença^(64,95,103)^.

No entanto, há situações em que o pai não encontra tempo para se envolver no cuidado ou ainda julga não possuir conhecimento e habilidades para cuidar do filho doente. Nesses casos, a mãe assume de modo hegemônico o cuidado do filho enfermo. O principal fator da falta de tempo paterno relaciona-se com os multipapéis que o pai assume, nos quais ele precisa dividir-se entre o trabalho remunerado para prover o sustento da família, cuidado com o filho doente, suporte e apoio para a companheira, responsabilidade com os outros filhos e afazeres domésticos. Dessa forma, a participação paterna no cuidado ao filho com doença grave é limitada^(88,103,104)^.

Com intuito de adquirir competência para exercer sua função paterna e os cuidados de que o filho doente necessita, o pai busca informações sobre a enfermidade, tratamento e cuidados direcionados à saúde da criança na internet, livros e televisão, sendo esse um mecanismo encontrado por ele para ajudar seu filho. Ressalta-se que quando o homem produz meios para atender as necessidades psicoemocionais e físicas do filho, o que compreende os cuidados com saúde, alimentação, higiene, proteção, apoio e afeto, ele cumpre um papel chamado de paternidade^(95,105,106)^.

O método canguru como estratégia de inserir o pai no cuidado ao filho hospitalizado em unidades de terapias neonatais tem sido amplamente discutido na literatura. Os resultados mostram que a posição canguru fortalece o laço afetivo entre a díade pai-filho, favorece uma maior participação paterna no processo de cuidar do neonato e ressignifica o ser pai, uma vez que possibilita ao homem viver a paternidade de modo completo, significativo e consciente do seu papel de cuidador. O pai, ao vivenciar esses benefícios, sente-se mais seguro em realizar os cuidados com o recém-nascido hospitalizado. Com isso, desperta o desejo de permanecer por maior tempo junto ao filho^(107,108,109,110,111,112)^.

Vale destacar que a unidade de terapia intensiva neonatal é um ambiente desafiador para os genitores. Para eles, ter um filho internado nesse serviço de saúde significa gravidade da doença e risco de morte. Altos níveis de estresse, sofrimento e medo de perder a criança são vivenciados pelos pais^(113)^.

Estudos acerca dos abalos psicológicos sofridos pelos pais frente à doença grave do filho revelam experiências dolorosas e assustadoras. Os genitores são acometidos por intenso desequilíbrio emocional que se expressa por meio do sofrimento, tristeza, ansiedade, angústia, desespero, desesperança, culpa, medo da morte, incertezas, frustração e incapacidade, sendo essa uma condição preocupante, desafiadora e desgastante para todos os integrantes da família^(114,115,116)^.

O pai também é afetado pela doença, hospitalização e tratamento do filho, vivenciando medo e pavor diante da possibilidade da morte da criança, frustração de ter um filho doente e com limitações, desespero e tristeza ao presenciar a condição de saúde em que a criança se encontra e inutilidade por não conseguir amenizar o sofrimento do filho. Contudo, o pai manifesta seus sentimentos de maneira diferente da mãe. Eles exteriorizam suas dores e preocupações por meio do choro e mostram-se irritados e nervosos, mas procuram não chorar na frente da mulher e da família, evitam falar sobre o problema e assumem um comportamento introspetivo, ficando mais calados do que de costume^(51,67)^.

O diagnóstico de uma doença grave no filho repercute em mudanças na dinâmica e rotina familiar, adaptações de papéis parentais e restrições na vida social e ocupacional dos pais. A doença e hospitalização da criança ocasiona prejuízo financeiro para a unidade familiar em virtude da diminuição das atividades laborais e até mesmo abandono do emprego, sendo que, na maioria das vezes, é a mulher quem abdica de sua vida profissional para se dedicar integralmente ao filho enfermo. Tal realidade reflete em baixa qualidade de vida e lazer para o homem, isolamento social, excesso de atribuições, falta de tempo e prioridade para o autocuidado e sobrecarga das responsabilidades financeiras para o pai. Além disso, tem-se o impacto negativo nas relações interpessoais familiares e conjugais^(117,118,119,120)^.

Diante da reorganização familiar para atender às necessidades da criança doente, o núcleo familiar enfrenta o distanciamento nas relações e ausência de convivência entre seus membros. O adoecimento do filho é visto pelos pais como um processo perturbador, permeado por infelicidade e estafa psicoemocional, tendo como resultado a ruptura da identidade familiar e matrimonial^(117,121,122)^.

Uma vida conjugal saudável requer equilíbrio físico e psicológico para que se instale uma íntima e amorosa relação entre o casal, o que não ocorre em pais de crianças doentes, visto que a enfermidade do filho é colocada como foco de atenção principal na vida dos progenitores. Sendo assim, a relação matrimonial dos pais é mediada por infelicidade, estresse e desarranjo emocional. Ademais, o adoecimento de um filho provoca nos pais esgotamento físico e mental, perda da privacidade, desinteresse sexual, conflitos e prejuízo na comunicação, contribuindo para o desgaste e separação conjugal^(123,124)^.

Considerando a importância de relevar a paternidade e encorajar a inserção do pai nos cuidados à criança com doença grave, julgamos colaborar de modo significativo com a comunidade acadêmica, visto que a formação dos profissionais de saúde e de enfermagem pouco explora o cuidado centrado na família e possui uma escassez de conhecimentos sobre figura paterna. Dessa forma, os resultados dessa revisão contribuem para o ensino, pesquisa e construção de um currículo integrado que incorpore em seus conteúdos de aprendizagem assuntos acerca da unidade familiar e do papel paterno com vista para formação profissional qualificada.

O presente estudo também contribui para a desconstrução sociocultural dos ideais de gênero no que se refere aos papéis parentais ao desvelar o sofrimento paterno nos casos em que o homem tem sua paternidade colocada à margem da vida do filho doente, aos fatores que contribuem para que o pai não participe ativamente dos cuidados ao filho com doença grave e aos benefícios que a presença paterna promove na saúde e desenvolvimento da criança, no bem-estar materno e na própria assistência prestada pelos profissionais de enfermagem.

Como limitação desta revisão de escopo, destaca-se a escolha por estudos apenas nos idiomas inglês, espanhol e português, a inclusão somente das dez primeiras páginas do Google Scholar e a indisponibilidade de texto completo de alguns artigos, o que pode ter prejudicado a coleta de dados e a seleção de trabalhos relevantes. Outro fator limitante foi o contexto pesquisado, visto que os temas cuidado paterno e igualdade de gêneros são pouco abordados na literatura científica, principalmente quando associados ao cuidado do pai ao filho com doença grave.

## CONCLUSÃO

Os resultados desta revisão possibilitaram mapear e descrever a participação paterna frente ao cuidado do filho com doença grave. Assim, verificou-se neste estudo que a participação do pai no cuidado ao filho com doença grave é limitada. Mesmo diante dos benefícios da inserção paterna nos cuidados com o filho doente, o pai enfrenta osbstáculos para exercer sua paternidade ativa relacionados com questões socioculturais e de gênero acerta dos papéis parentais. Ademais, constatou-se que ser pai de um filho com doença grave repercute negativamente na vida do homem causando impactos econômicos, familiares e psicoemocionais.

Salientam-se lacunas identificadas na formação acadêmica de profissionais da saúde sobre a paternidade e cuidado centrado na família, evidenciando a importância de capacitação, ainda durante a graduação, para atuação na atenção à saúde da criança, pois muitos profissionais deparam-se com estigmas históricos, socioculturais e de gênero relacionados aos papéis parentais, repercutindo em uma assistência desumana e fragmentada para a tríade mãe, pai e filho e, como consequência, limitando o exercício da paternidade.

Por fim, vale dizer que novas pesquisas têm de ser realizadas com o intuito de investigar questões que ainda não foram suficientemente exploradas e com aprimoramentos metodológicos necessários para avançar na compreensão acerca das repercussões socioculturais e de gênero que influenciam e limitam o exercício da paternagem, do papel paterno e os benefícios da presença paterna e da inserção do pai nos cuidados ao filho com doença grave.

Ademais, estudos futuros devem ambicionar fornecer conhecimento verticalizado sobre a paternidade aos profissionais de saúde, pesquisadores e formuladores de políticas públicas, com o intuito de subsidiar a incorporação de estratégias na prática assistencial e nos serviços de saúde infantojuvenil, para melhorar a experiência paterna frente ao cuidado do filho com doença grave, envolver a figura paterna nas relações familiares, intensificar a humanização da assistência para unidade familiar, promovendo ações que fortaleçam o vínculo pai e filho e revelem a paternidade ativa.

# Translation: La participación del padre en el cuidado de su hijo con una enfermedad grave: revisión del alcance

**DOI:** 10.1590/1980-220X-REEUSP-2024-0278es

**Authors:** Rafaela Sterza da Silva, Cinara Bozolan Coppo, Edrian Maruyama Zani, Adriana Valongo Zani Zani

**Affiliations:** 1 Universidade Estadual de Londrina, Programa de Pós-graduação em Enfermagem, Londrina, PR, Brasil.

**Keywords:** Padre, Relaciones Padre-Hijo Conducta Paterna, Cuidado del Niño, Enfermedad Catastrófica

## Abstract

**Objetivo:**

Mapear y describir estudios existentes en la literatura sobre la participación de los padres en el cuidado de niños con enfermedades graves.

**Método:**

Revisión de alcance según metodología JBI. La búsqueda se llevó a cabo en octubre 2023, con estudios publicados íntegramente en portugués, inglés o español, sin limitaciones de tiempo e indexados en bases de datos y portales de tesis y disertaciones. Dos revisores leyeron el título y el resumen de los estudios y exportaron los datos a una hoja de cálculo *Microsoft Excel®* para agrupar y eliminar duplicados. Los datos se sintetizaron en forma escrita y visual, utilizando diagramas.

**Resultados:**

La búsqueda resultó en 961 estudios, 38 estudios siendo elegibles para componer la muestra. El análisis de los estudios permitió identificar que la participación paterna en el cuidado de un niño con enfermedad grave implicó beneficios para el niño, la madre y la familia; las formas de cuidado paterno; el papel social del padre; los impactos psicoemocionales, socioeconómicos y familiares y las barreras de género y socioculturales respecto de la figura paterna.

**Conclusión:**

La participación paterna en el cuidado de un niño con una enfermedad grave resultó ser un fenómeno complejo que requiere la deconstrucción de estigmas históricos, socioculturales y de género relacionados con los roles parentales.

## INTRODUCCIÓN

Debido a influencias políticas, económicas, sociales y religiosas, la unidad familiar ha sufrido grandes cambios a lo largo de los años en cuanto a su concepto, estructura, dinámica y el papel que desempeña cada miembro. La familia se entiende como la base de la organización de una sociedad y, durante siglos, se construyó sobre el patriarcado, en el que las mujeres no tenían voz ni voto y sólo eran responsables del cuidado de la casa y la educación de los hijos, mientras que los hombres tenían el papel de autoridad y proveedor de la familia^(1)^.

El papel del padre en la sociedad contemporánea ha evolucionado considerablemente, yendo más allá del mero papel de proveedor económico y autoridad familiar. Hoy en día, los padres se implican cada vez más en el cuidado y la educación de sus hijos, desempeñando un papel activo en la transmisión de valores, afecto y protección^(2)^. Esta transformación de la paternidad se refleja en la resignificación del concepto de padre, que ahora se ve como protagonista en la crianza de los hijos, buscando establecer vínculos afectivos profundos y participar activamente en la vida cotidiana familiar^(3,4)^.

Sin embargo, la sociedad sigue manteniendo patrones tradicionales que a menudo relegan al padre a un papel secundario en la crianza de los hijos, especialmente en contextos de cuidado. La figura paterna, a pesar de su deseo de mayor implicación, se enfrenta a obstáculos culturales y sociales que asocian la crianza principalmente a la figura materna^(5,6)^. Esta visión tradicional limita la implicación paterna, haciendo que muchos padres sientan que están apoyando el proceso de crianza y cuidado de sus hijos^(7)^.

Esta dualidad de papeles se agrava en situaciones de enfermedad infantil, en las que la figura paterna tiende a volver a su papel de proveedor de la familia, lo que refleja el conflicto entre la concepción moderna de la paternidad y la ideología retrógrada de la masculinidad. En este contexto, muchos padres siguen sintiéndose presionados para asumir las responsabilidades económicas de la familia, mientras que el cuidado directo del niño enfermo se centra en las madres, perpetuando los estigmas de género arraigados en la sociedad^(8,9)^.

La literatura científica subraya la importancia de la implicación paterna en el cuidado de los niños, especialmente en casos de enfermedad grave. Los estudios demuestran que la participación activa de los padres en el cuidado de sus hijos enfermos puede tener un impacto positivo en el desarrollo del niño y mejorar la calidad de los cuidados prestados^(10,11)^. Sin embargo, la participación activa del padre no es un consenso entre los estudiosos, lo que demuestra que muchos padres todavía operan bajo concepciones tradicionales de género, limitando el ejercicio de la paternidad^(12)^.

Aunque la participación de los padres en el cuidado de los niños enfermos es controvertida, la literatura tampoco ofrece una definición precisa de enfermedad infantil grave. Sin embargo, establece una lista de enfermedades caracterizadas por su desarrollo progresivo y duradero, con potencial para causar discapacidad, dependencia de cuidados y amenaza de muerte, destacando la complejidad de las condiciones que requieren cuidados continuos e intensivos^(13)^. En el contexto de este estudio, la enfermedad grave se definió como una condición clínica con el potencial de causar graves consecuencias para la salud, que pueden conducir a una discapacidad significativa, complicaciones graves o incluso la muerte. Estas enfermedades suelen requerir una hospitalización prolongada debido a la necesidad de numerosas intervenciones médicas.

Durante el tratamiento y la rehabilitación de estos niños, la participación paterna suele ser mínima o incluso inexistente, lo que pone de manifiesto la urgente necesidad de una mayor concienciación y apoyo para promover la equidad en la participación de los padres con niños con enfermedades graves^(14)^.

Estudios han mostrado avances en el conocimiento sobre la paternidad, las influencias socioculturales y de género en el ejercicio de la paternidad, el papel paterno en la relación padre-hijo y la función paterna en el desarrollo infantil, demostrando el reciente interés de los investigadores por la figura paterna^(15,16,17,18,19,20,21)^. Sin embargo, poco se sabe sobre el ejercicio de la paternidad ante un hijo gravemente enfermo. En una búsqueda realizada en el*Open Science Framework y en*el*Registro prospectivo internacional de revisiones sistemáticas*(PROSPERO), no se identificaron revisiones de alcance ni revisiones sistemáticas existentes que respondieran a la pregunta: ¿cómo participa el padre en el cuidado de un hijo con una enfermedad grave?

A raíz de este escenario, con el objetivo de dar a conocer la figura paterna y contribuir a la consolidación del conocimiento sobre el cuidado paterno de niños con enfermedades graves, el objetivo de esta investigación fue mapear y describir los estudios existentes en la literatura sobre la participación de los padres en el cuidado de niños con enfermedades graves.

## MÉTODO

### Diseño del Estudio

Se trata de una revisión de alcance que buscaba explorar, mapear y sintetizar la evidencia científica e identificar lagunas en el conocimiento sobre la presencia y participación de los padres en el cuidado de niños con enfermedades graves. Este estudio se desarrolló de acuerdo con el manual del JBI^(22)^y las directrices de la*Lista de Verificación y Explicación*de*los Elementos Preferidos para la Elaboración de Informes de Revisiones Sistemáticas y Metaanálisis*(PRISMA-ScR) para la transparencia en la redacción del informe de revisión^(23)^.

El protocolo de esta revisión se registró en el*Open Science Framework*(OSF), con la identificación https://osf.io/5fzrg/DOI 10.17605/OSF.IO/5FZRG.

Para la elaboración de esta revisión se siguieron las siguientes cinco etapas^(24)^: 1) identificación de la pregunta de investigación; 2) identificación de los estudios relevantes; 3) selección y evaluación inicial de los estudios; 4) análisis de los datos; y 5) agrupación, síntesis y presentación de los datos.

### Recogida de Datos

Para formular la pregunta de investigación (etapa 1), se utilizó la nemotecnia PCC (Población, Concepto y Contexto), descrita en cuadro 1.

**Cuadro 1:** Estrategia PCC (Población, Concepto y Contexto) – Londrina, PR, Brasil, 2024.

	PCC	Descripción
P	Población	Padre
C	Concepto	Estudios que aborden el cuidado paterno
C	Contexto	Cuidado de niños, Enfermedad grave o catastrófica

A partir de esta estrategia, se elaboró la pregunta de investigación: ¿Cómo participa el padre en el cuidado de su hijo con una enfermedad grave?

La identificación de estudios relevantes (etapa 2) implicó la selección de bases de datos, la definición de estrategias de búsqueda basadas en descriptores y operadores booleanos, y la formulación de criterios de inclusión y exclusión. La búsqueda se realizó en las siguientes bases de datos CINAHL (*Cumulative Index to Nursing and Allied Health Literature*), Embase (*Excerpta Medica dataBASE*),*Scopus*,*Web of Science*, SciELO (*Scientific Electronic Library Online*), PubMed y la Biblioteca Virtual de Salud (BVS). Para investigar la literatura gris se utilizaron*Google Scholar*y el Catálogo de Tesis y Disertaciones de la Coordinación para el Perfeccionamiento del Personal de Enseñanza Superior (CAPES). Los estudios se recuperaron de las primeras 30 (treinta) páginas de Google*Scholar*. Cabe señalar que las estrategias de búsqueda se ajustaron de acuerdo con las particularidades de cada base de datos.

Los datos fueron recolectados en octubre de 2023 a través del portal de revistas de la Coordinación para el Perfeccionamiento del Personal de Enseñanza Superior (CAPES), vía Comunidad Académica Federada (CAFe). Se utilizó la función «búsqueda avanzada», utilizando los descriptores MeSH (*Medical Subject Headings*) y DeCS (Descriptores de Ciencias de la Salud) y los operadores booleanos*AND*y*OR*. En el Catálogo CAPES de Tesis y Disertaciones, la estrategia de búsqueda se aplicó para el término solitario y el cruce de términos sin operadores booleanos. La estrategia consistió en cruzar los términos entre sí, como se muestra a continuación:

- Descriptores de Ciencias de la Salud (DeCS): Father*AND*Father-Child Relations*OR*Paternal Behaviour*AND*Childcare*AND*Serious Illness.
- *Medical Subject Headings*(MeSH):*Fathers AND Father-Child Relations OR Paternal Behaviour AND Child Care AND Critical Illness.*

Se incorporaron los descriptores no controlados «niño enfermo» y «cuidado del padre». En el Cuadro 2 se detalla la estrategia de búsqueda utilizada en cada base de datos.

**Cuadro 2:** Elaboración de la estrategia de búsqueda en las diversas bases de datos/portales – Londrina, PR, Brasil, 2024.

Base de datos	Estrategia de búsqueda
BVS	#1 Pai *AND* Relações Pai-Filho *OR* Comportamento Paterno *AND* Cuidado da Criança *AND* Doença Grave; #2 Pai *AND* Cuidado da Criança *AND* Doença Grave; #3 Pai *AND* Cuidado da Criança *AND* “Filho doente”; #4 Comportamento Paterno *AND* Filho Doente *AND* Doença Grave
CINAHL	#1 *Fathers AND Father-Child Relations OR Paternal Behavior AND Child Care AND Critical Illness*; #2 *Fathers AND Child Care AND Critical Illness*
PubMed	#1 *Fathers AND Father-Child Relations AND Child Care AND Critical Illness*; #2 *Fathers AND Child Care AND Critical Illness*; #3 *Paternal Behavior AND Child Care AND Critical Illness*; #4 *Paternal Behavior AND “Sick Child” AND Critical Illness*; #5 *Paternal Behavior AND “Sick Child”*
Embase	#1 *Father AND Father child relation OR Paternal Behavior AND Child Care AND Critical Illness*; #2 *Father AND Child Care AND Critical Illness*; #3 *Father child relation OR Paternal Behavior AND Child Care AND “Sick Child”*; #4 *Father child relation OR Paternal Behavior AND “Sick Child”*; #5 *Father AND Child Care*
Scopus	#1 *Fathers AND Father-Child Relations AND Child Care AND Critical Illness*; #2 *Father AND Child Care AND Critical Illness*; #3 *Paternal Behavior AND “Sick Child”*
*Web of Science*	#1 *Fathers AND Father-Child Relations OR Paternal Behavior AND Child Care AND Critical Illness*; #2 *Fathers AND Child Care AND Critical Illness*; #3 *Paternal Behavior AND “Sick Child”*; #4 *Fathers AND Child Care AND “Sick Child”*
*SciELO*	#1 *Fathers AND Father-child relation OR Paternal Behavior AND Child Care AND Critical Illness*; #2 *Fathers AND Child Care AND Critical Illness*; #3 *Father-child relation AND “Sick Child*”; #4 *Fathers AND “Sick Child” AND Critical Illness*; #5 *Fathers AND Child Care*; #6 *Fathers AND “Sick Child”*
Google acadêmico	#1 Pai *AND* Relações Pai-Filho *OR* Comportamento Paterno *AND* Cuidado da Criança *AND* Doença Grave
CAPES	#1 “Cuidado do Pai”; #2 “Filho Doente”; #3 Relações Pai-Filho; #4 Comportamento Paterno

### Criterios de Selección

Los criterios de elegibilidad de los estudios se establecieron con el fin de asegurar que sólo los documentos pertinentes al ámbito de la revisión fueran incluidos en el análisis, garantizando la obtención de una revisión de alta calidad, apoyada en evidencias relevantes sobre la presencia y participación de los padres en el cuidado de niños con enfermedades graves.

Esta revisión incluyó estudios publicados en bases de datos establecidas y literatura gris que involucraban a los padres como población y abordaban el concepto de comportamiento paterno, la relación padre-hijo y la participación paterna en el cuidado de los hijos en el contexto de enfermedades graves o catastróficas.

Así, los criterios de elegibilidad fueron: publicaciones sin marco temporal, en portugués, inglés y español, de cualquier diseño metodológico, disponibles en su totalidad y gratuitamente en medios electrónicos, así como disertaciones y tesis que respondieran a la pregunta de investigación. Se excluyeron los editoriales, las cartas de respuesta y los artículos de opinión, y los documentos duplicados sólo se contaron una vez.

### Extracción y Análisis de Datos

La selección y evaluación inicial de los estudios (etapa 3) implicó la participación de dos revisores independientes que, en primer lugar, realizaron una lectura exhaustiva del título y el resumen de los estudios encontrados. A continuación, los datos de los estudios elegibles se exportaron a una hoja de cálculo*de Microsoft Excel®*para su agrupación y eliminación de duplicados. Por último, los revisores leyeron los materiales en su totalidad, aplicando de nuevo los criterios de elegibilidad para la posterior extracción de datos.

Se investigaron las citas de todos los estudios que cumplían los criterios de inclusión para identificar estudios adicionales. Los estudios elegidos en esta nueva investigación se analizaron del mismo modo que los demás, como se ha mencionado anteriormente, y se identificaron por separado en la presentación de los resultados.

Los desacuerdos entre los investigadores durante el proceso de selección de los estudios fueron resueltos por un tercer revisor experto en el tema, que proporcionó la opinión final. Los resultados cuantitativos de cada base de datos, los estudios excluidos y sus razones, el total de estudios incluidos para el análisis y la síntesis de datos, se describieron mediante un diagrama de flujo.

El análisis de datos (etapa 4) consistió en analizar y categorizar los datos extraídos para identificar patrones, tendencias y lagunas en la bibliografía. Los estudios fueron analizados por dos autores independientes y se utilizó un cuadro sinóptico, elaborado por los revisores, para sistematizar los datos extraídos, que constaba de la siguiente información: identificación del estudio, nombre de los autores, año de publicación, título, tipo de publicación, país de origen, revista, tipo de estudio, objetivo del estudio y principales resultados y consideraciones.

Cabe señalar que el cuadro sinóptico para la recogida de datos fue previamente evaluado por los autores mediante una prueba piloto. La prueba piloto para la extracción de datos fue realizada por dos investigadores, que utilizaron la misma estrategia de búsqueda mencionada anteriormente y probaron el cuadro sinóptico en 10 estudios al azar para alinear los criterios de elegibilidad y asegurarse de que se incluían todos los datos relevantes. Tras la búsqueda piloto en las bases de datos y la prueba del marco sinóptico, los dos investigadores responsables de la prueba piloto se reunieron con los demás autores para discutir y evaluar la aplicabilidad del marco sinóptico, la estrategia de búsqueda y si la metodología empleada permitía alcanzar los objetivos de la investigación. Cabe señalar que, tras la discusión entre los investigadores, no hubo necesidad de reformular el proceso de búsqueda y extracción de datos.

Los datos se agruparon, resumieron y presentaron (etapa 5) de forma escrita y visual, mediante diagramas, con el objetivo de visualizar y comprender los resultados. En consonancia, se realizó un análisis crítico de los resultados de forma descriptiva y reflexiva de las evidencias obtenidas.

### Aspectos Éticos

La investigación se llevó a cabo de acuerdo con los principios éticos y las directrices metodológicas adecuadas, cumpliendo con las buenas prácticas en la investigación científica y respetando los derechos de autor de los estudios citados. Al tratarse de un estudio de revisión basado en el análisis de datos secundarios de acceso público, y al no implicar directamente la participación de seres humanos, no hubo cuestiones éticas que requirieran la consideración de un comité de ética.

## RESULTADOS

La búsqueda dio como resultado 961 estudios, de los que se eliminaron 135 publicaciones por duplicación. Así, se analizaron 826 estudios en base a sus títulos y resúmenes, excluyéndose 694 documentos por no cumplir el tema, por no estar disponible el texto completo y por tratarse de estudios de revisión, quedando 132 documentos para la evaluación completa de los criterios de elegibilidad. De éstos, se excluyeron 96 estudios, lo que dio lugar a la selección de 36 documentos. Cabe señalar que se añadieron dos publicaciones de la lista de referencias, con lo que la muestra final quedó constituida por un total de 38 estudios.

La Figura 1 ilustra el diagrama de flujo del proceso de selección de estudios de acuerdo con las recomendaciones del JBI, adaptadas de la*Lista de Comprobación de los Elementos de Información Preferidos para Revisiones Sistemáticas y Metaanálisis*(PRISMA-ScR)^(25)^.

**Figura 1:** Flujograma de selección de estudios adaptado de PRISMA-ScR^(25)^ – Londrina, PR, Brasil, 2024.

Los 38 estudios seleccionados para componer los resultados de esta investigación se clasificaron y organizaron en una secuencia lineal de tiempo creciente, según el año de publicación, el tipo de publicación, el país de origen y la base de datos (Figura 2). Cabe destacar que las citaciones de la Figura 2 fueron listadas secuencialmente de acuerdo con el orden de las bases de datos utilizadas en la estrategia de búsqueda: BVS^(26,27)^, CINAHL^(28,29,30,31)^, Embase^(32,33,34,35)^, PubMed^(36)^, SciELO^(37^–^41)^, Scopus^(42)^,*Web of Science(* ^43,44)^, CAPES^(45)^,*Google Scholar* ^(46^–^61)^y búsquedas de citaciones/otras fuentes^(62,63)^.

**Figura 2:** Cronología de los estudios seleccionados según el año de publicación, el tipo de publicación, el número de publicaciones según el país de origen y el porcentaje de publicaciones según el año – Londrina, PR, Brasil, 2024.

Los años de mayor producción científica sobre el tema fueron 2013^(36,41,50,56,59)^ y 2020^(34,35,43,54,55)^, totalizando cinco estudios cada uno, seguidos de 2019^(32,51,52,60)^ (n = 4); 2010^(30,31,53)^, 2012^(29,42,49)^ y 2018^(26,48,58)^ con tres publicaciones cada uno; 2014^(40,45)^, 2016^(28,47)^, 2017^(27,38)^ y 2022^(37,61)^ con dos estudios cada año. Los años con una sola publicación fueron 1991^(33)^, 2004^(46)^, 2006^(57)^, 2008^(62)^, 2009^(44)^, 2015^(63)^y 2021^(39)^.

La mayoría de las publicaciones fueron artículos científicos^(26,27,28,29,30,31,32,33,34,35,36,37,38,39,40,41,42,43,44,46,47,48,49,50,51,52,58,62,63)^ (n = 29). Las disertaciones^(45,53,54,55,56,57)^ representaron el 15,7% de los estudios y sólo hubo una producción de trabajo de fin de curso^(60)^ (TCC), monografía^(59)^ y tesis^(61)^.

Los países de origen de los estudios fueron variados, siendo Brasil^(26,27,28,29,30,31,38,39,40,41,42,46,47,48,50,51,52,53,54,55,56,57,58,59,60,61,62,63)^ el país que más estudios ha desarrollado sobre el tema (n = 28), seguido de Irlanda del Norte^(44,49)^ (n = 2), Colombia^(37,45)^ (n = 2), Inglaterra^(33)^(n = 1), Suiza^(34)^(n = 1), Etiopía^(35)^(n = 1), México^(43)^(n = 1), Mali^(36)^(n = 1) y Polonia^(32)^(n = 1).

El análisis de los estudios nos permitió identificar que la participación paterna en el cuidado de un hijo con una enfermedad grave implica tres categorías: 1. Participación del padre en el cuidado de un hijo con una enfermedad grave, en la que se analizan los beneficios de la participación paterna, las formas de cuidado y el papel paterno con el hijo enfermo, la unidad familiar y la madre; 2. Repercusiones de tener un hijo enfermo; y 3. El papel paterno en el cuidado de un hijo con una enfermedad grave. 2. Repercusiones de tener un hijo enfermo, que dilucida los impactos psicoemocionales, socioeconómicos y familiares experimentados por el padre; 3. Obstáculos al cuidado paterno, que retrata las barreras de género y socioculturales que rodean a la figura paterna.

A continuación se resumen los principales resultados de los estudios seleccionados (figura 3).

**Figura 3:** Mapa mental de los principales resultados de los estudios incluidos en la revisión del alcance – Londrina, PR, Brasil, 2024. **Fuente de la imagen:** br.freepik.com

Los resultados apuntaron a barreras socioculturales para la participación paterna en el cuidado de un niño con una enfermedad grave, siendo el cuidado de un niño gravemente enfermo un papel asignado a las mujeres. Cuando su hijo enferma, la madre deja su trabajo, su casa, su marido, sus otros hijos y su vida social para dedicarse a su hijo con enfermedad grave, mientras que el padre asume el papel de coadyuvante en esta actividad. Existe una tendencia materna a creer que nadie es capaz de cuidar de su hijo, es decir, para la madre, sólo ella está capacitada para esta actividad.

En cuanto a las barreras de género, los estigmas sociales machistas en los que han sido socializados los hombres sugieren que los padres no están capacitados para cuidar de sus hijos. La necesidad de reincorporarse al trabajo, la concepción arcaica de la masculinidad en la que el padre es el proveedor y la madre la cuidadora, la falta de experiencia y capacidad para atender a un hijo con una enfermedad grave hacen que la madre sea considerada por los padres como la persona más capacitada para reconocer la enfermedad y atender al niño enfermo. Además, el prejuicio contra el género masculino asociado a la estructura de las salas colectivas dificulta la estancia de los padres con sus hijos, ya que la presencia conjunta del padre de un niño con la madre de otro en la misma unidad sería inviable, convirtiendo la figura paterna en un visitante y limitando su participación en el cuidado del niño enfermo.

En cuanto al impacto familiar, psicoemocional y socioeconómico, se observó que un hijo gravemente enfermo causa daños en la dinámica familiar, con repercusiones en términos de funcionamiento familiar de baja calidad, división de los roles parentales, ruptura psicológica y conyugal. Para los padres, las mayores dificultades relacionadas con el cuidado de un hijo con una enfermedad grave son económicas, profesionales, sociales y emocionales, la falta de tiempo para el autocuidado, los elevados costes del tratamiento y la falta de apoyo de otros miembros de la familia. La experiencia de tener un hijo gravemente enfermo está impregnada de trastornos psicológicos y emocionales como ansiedad, depresión, estrés, miedo, angustia, inseguridad y frustración.

En cuanto al cuidado paterno, los estudios revelaron que cuando un niño enferma, el cuidado paterno directo del niño es limitado, ya que el padre cree que carece de las habilidades y los conocimientos necesarios para cuidar del niño. Además, la escasa participación paterna en el cuidado de un hijo con una enfermedad grave está relacionada con las actividades laborales del hombre. Por otro lado, los padres encuentran otras formas de mantener el vínculo y participar en el cuidado de sus hijos hospitalizados y gravemente enfermos, como el contacto piel con piel (posición canguro), el tacto, las caricias, las visitas al hospital, hablar con sus hijos y expresar sentimientos positivos. En el contexto doméstico, los cuidados paternos están relacionados con la búsqueda de información sobre la enfermedad del niño para promover cambios positivos en su estado de salud, el sueño y el descanso, la alimentación, la higiene y el ocio con momentos de interacción lúdica, como el juego.

En cuanto al papel paterno, se observó que la idea de paternidad ha cambiado con el tiempo. Los hombres de hoy se implican más en el cuidado de sus hijos que sus antepasados. Como forma de ser útil y participar en el cuidado de su hijo gravemente enfermo, el padre asume funciones importantes con la madre, el niño y la unidad familiar. Con respecto a la madre, el padre desempeña el papel de apoyo emocional, apoyo materno para hacer frente a la enfermedad del niño y consuelo, fuerza y afecto ante la hospitalización del niño, ofreciendo momentos de descanso materno y animando a su pareja a salir del entorno hospitalario durante un breve periodo de tiempo. Con el niño, el papel paterno se centra en proporcionar apoyo emocional, ser paciente, dedicado y mostrar amor y afecto, haciendo que el niño se sienta seguro y aliviando sus experiencias dolorosas. Además de ser el sostén y cabeza de familia, el papel del padre con la unidad familiar incluye garantizar el bienestar, proteger y apoyar a los demás miembros de la familia y asumir las tareas domésticas, incluido el cuidado de los demás niños en casa.

En cuanto a los beneficios de la participación paterna en el cuidado de un niño con una enfermedad grave, los estudios incluidos mostraron que los padres reconocen la importancia de participar en la crianza de sus hijos y que su papel es fundamental para el desarrollo del niño. De este modo, la participación del padre compartiendo los cuidados de un niño gravemente enfermo contribuye directamente a la calidad de la atención prestada por el equipo sanitario, y se considera una experiencia beneficiosa y positiva. El padre presente transmite amor, calor y seguridad al niño y aumenta la intimidad con su hijo, dándole la oportunidad de reforzar su papel.

## DISCUSIÓN

A lo largo de la historia, el papel del hombre en el cuidado de los hijos ha evolucionado considerablemente. A principios del siglo XX, la paternidad se consideraba un deber secundario frente al trabajo remunerado. Sin embargo, desde los años 50 y 60, el comportamiento en relación con la paternidad ha cambiado, en gran parte debido a la progresiva inclusión de la mujer en el mercado laboral. Como consecuencia, los padres han pasado a desempeñar un papel importante en la crianza de los hijos y, en muchas culturas occidentales, la figura paterna se ha implicado activamente en el cuidado de los niños. A pesar de los avances sociales, sigue existiendo desigualdad en el papel de hombres y mujeres en la vida cotidiana de las familias de niños con enfermedades graves, siendo la madre quien asume la mayor parte de las tareas de cuidado^(64,65)^.

Martins y Reis^(66)^, en sus estudios, revelaron que las madres se consideran las principales cuidadoras de sus hijos, destacando la poca participación paterna. Para ellas, aunque se considere que el padre está presente en la crianza de los hijos, cuando el niño cae enfermo, en lo que se refiere a los cuidados relacionados con la enfermedad, el hombre no desempeña ningún papel. Es más, la madre atribuye el cuidado del niño enfermo como un papel hegemónicamente maternal.

De este modo, no se comparte el cuidado parental del niño institucionalizado, ya que la madre permanece con su hijo en todo momento, limitando la participación paterna. Esta decisión se basa en la percepción de la madre de que los niños están más tranquilos en su presencia. Además, la madre no considera que el padre pueda ocuparse del niño enfermo, especialmente en situaciones de enfermedad grave, tan bien como ella^(67)^.

Durante décadas, los padres han sido pasivos a la hora de cuidar de sus hijos, y este papel está culturalmente reservado a las mujeres que, desde una edad temprana, ya están orientadas y nutridas hacia el universo materno, dejando a los hombres pocas oportunidades de desarrollar la paternidad. Dado que la responsabilidad del cuidado se atribuye socialmente al papel de la mujer en la sociedad y se reproduce entre generaciones, es importante deconstruir las representaciones sociales e identitarias del modelo paterno retrógrado y revelar una nueva figura paterna^(68,69)^.

Este proceso de continua transformación y reconstrucción, que se refleja en los nuevos conceptos de familia, ha reestructurado lo que antes se llamaba el padre proveedor en lo que hoy queremos: una paternidad corresponsable de la reproducción y crianza de sus hijos^(70)^. De este modo, la paternidad no se limita a la disciplina y a la provisión de recursos económicos, sino también a la participación en el cuidado diario desde la primera infancia hasta la vida escolar^(71)^.

Este cambio de paradigma exige que los padres tengan conocimientos sobre el cuidado de los hijos para que puedan desempeñar su papel de forma independiente, segura y eficaz, lo que redundará en beneficios para el desarrollo biopsicosocial de sus vástagos^(72)^.

Sin embargo, la realidad es controvertida. Los autores señalan que el deber de volver al trabajo y la vieja idea del hombre como «sostén de la familia» y la madre como cuidadora, así como el miedo y la falta de experiencia y capacidad del padre para cuidar de su hijo enfermo, hacen que la madre sea vista por el padre como la más capacitada para cuidar del niño enfermo. Además, el padre se siente responsable de proveer a las necesidades materiales de la familia y, en consecuencia, no se implica directamente en el cuidado del niño, asumiendo el papel de apoyo materno^(66,73)^.

Cabe señalar que existen debilidades legales en relación al rol paterno, en que los lugares de trabajo no reconocen la paternidad y no garantizan los derechos garantizados en las diversas políticas públicas que incentivan la participación de los padres en el desarrollo de sus hijos, como la ley de acompañamiento, la licencia de paternidad y el Estatuto del Niño y del Adolescente^(74,75)^.

Asimismo, en cuanto a la falta de garantías en el mercado laboral, está la informalidad del trabajo, ya que muchos trabajadores por cuenta propia o informales no tienen una vida financiera estable, lo que hace que el hombre, incluso ante la enfermedad de su hijo, mantenga sus actividades laborales para pagar sus obligaciones como jefe de familia. Así, la informalidad y las largas jornadas de trabajo desafían el ejercicio de la paternidad^(76)^.

La literatura muestra otros factores que dificultan la promoción de la paternidad activa relacionada con los servicios de salud, como la feminización del cuidado de los hijos, la invisibilización paterna, la corta licencia de paternidad, la idealización de un padre que es soporte de la madre, la segregación de género en el rol parental y las características de las instituciones hospitalarias. Estas adversidades contribuyen a la escasa participación paterna en el cuidado de los niños enfermos y a la poca aceptación de los padres en las instituciones sanitarias^(74,77)^.

El ámbito hospitalario es un espacio de difícil incorporación de los padres, pues además de no reconocer la paternidad, la infraestructura física no proporciona la acogida y el confort para su inclusión. La disimetría en el reconocimiento de las figuras parentales por el equipo de salud contribuye al distanciamiento de los padres. Esta representación estrictamente materno-filial del cuidado de los niños debe ser rota por los profesionales de la salud, centrándose en la familia^(67,78,79)^.

A su vez, los profesionales sanitarios reconocen la importancia de compartir los cuidados con la familia y de implicar a la figura paterna en el cuidado del niño compartiendo las responsabilidades parentales. Sin embargo, existe un desconocimiento sobre el significado real y las formas de abordar y aplicar una atención centrada en la familia y en el padre durante el proceso de hospitalización de un niño con una enfermedad grave^(80,81)^.

En general, los profesionales han sido formados para dirigir la orientación y la atención al cuidador principal, es decir, al que está presente la mayor parte del tiempo durante la hospitalización del niño. Cuando se les pregunta quién acompaña al niño en el entorno hospitalario, los profesionales afirman que es la madre, mientras que el padre está ausente en ese momento. Para ellos, el cuidador principal del niño enfermo ya ha sido preestablecido por la unidad familiar y, por lo general, la persona que asume este papel es la que no trabaja o ha dejado de trabajar para dedicarse exclusivamente al niño enfermo, es decir, la madre. En consecuencia, la implicación paterna en el cuidado del niño es menos significativa que la de las madres, lo que dificulta la creación de un vínculo entre los profesionales y los padres, lo que provoca que la paternidad no sea reconocida por el equipo sanitario^(82,83,84)^.

Los estudios demuestran que la mujer, como principal responsable del cuidado de los niños con enfermedades graves, se enfrenta a cambios drásticos en su rutina y estilo de vida, lo que provoca un desequilibrio psicoemocional. La gran cantidad de tiempo invertido en el cuidado del niño enfermo, unido a la ausencia del padre, hace que las madres experimenten soledad, privación de sueño y se sientan abrumadas. La madre tiende a priorizar el cuidado del niño a expensas de su propia vida personal, por lo que están cansadas, deprimidas, estresadas y tienen una mala calidad de vida^(85,86,87,88)^.

Ante esto, se puede inferir la importancia de la participación del padre en el cuidado del hijo enfermo, y su implicación es beneficiosa para la salud materna, ya que el padre presente sirve de apoyo psicoemocional a la madre y fortalece el trinomio padre/madre e hijo en un reparto saludable de las responsabilidades parentales, proporcionando una experiencia materna saludable en el afrontamiento de la enfermedad del hijo con enfermedad grave^(89,90)^.

Los beneficios de la participación paterna en el cuidado de un hijo enfermo trascienden los relacionados con la madre, y también aportan mejoras en el estado general del niño. Los padres participativos promueven el bienestar emocional de sus hijos, reduciendo el estrés y favoreciendo el desarrollo cognitivo del niño^(91,92)^.

La literatura atribuye ventajas al desarrollo biopsicosocial del niño relacionadas con la presencia paterna. Entre ellas, la consolidación del vínculo y mayor calidad en la relación padre-hijo, lo que favorece la formación de la identidad del niño; el apoyo emocional y la promoción de la resiliencia infantil ante la adversidad y el fortalecimiento de los vínculos afectivos, lo que repercute en un niño más seguro, con mayor autoestima, empatía y solidaridad. Además, la presencia de una figura paterna contribuye al tratamiento y a la recuperación del niño, reduciendo la duración de la hospitalización^(83,93,94)^.

Cuando experimenta la enfermedad de su hijo, el hombre reafirma su papel paterno, deseando no sólo cumplir la función de proveedor de la familia, sino también mostrar interés por implicarse en todos los sentidos en el cuidado del niño. El nuevo padre asume el papel de cuidar de la madre, del niño enfermo, de la casa y de la familia^(95)^.

En cuanto a la figura materna, el padre cumple su papel estando atento a las necesidades psicoemocionales de la mujer, acogiendo sus miedos e inseguridades, proporcionándole apoyo y aliviando su agotamiento físico, mental y psicológico. En sus momentos más angustiosos y dolorosos, las palabras de consuelo y esperanza pronunciadas por los padres sirven de fuerza y aliento, ayudando a la madre a afrontar la enfermedad de su hijo de forma serena, confiada y optimista^(96,97)^.

Las investigaciones sobre la paternidad confirman que la figura paterna tiene un impacto positivo en las habilidades sociales del niño, además de promover el bienestar psicosocioemocional, ayudar a la autoconfianza del niño, mejorar su rendimiento escolar y reducir los problemas de comportamiento^(98,99,100)^.

Con un niño enfermo, el padre desempeña su papel protegiendo y garantizando el desarrollo del niño. Corresponde al padre ser afectuoso, atento, acogedor, mostrar seguridad, minimizar el sufrimiento del niño y participar en el cuidado del niño institucionalizado^(101,102)^.

En el contexto familiar, el papel del padre va más allá del entorno hospitalario, y es responsable de mantener la integridad y el funcionamiento de la unidad familiar. Sus obligaciones se orientan hacia el apoyo y el cuidado de los demás miembros de la familia, como el apoyo y el cuidado de los demás niños en casa y la realización de las tareas domésticas^(67,73,79)^.

Los estudios también apuntan a una transición en el rol paterno, revelando que los padres se preocupan cada vez más por participar en el cuidado de los niños con enfermedades graves, procurando acompañar a sus hijos a las consultas médicas y estar presentes durante el proceso de enfermedad y tratamiento. En este sentido, los padres se implican en el cuidado emocional y físico de sus hijos, realizando diversos cuidados, entre los que se incluyen la creación de un vínculo afectivo, la educación, el ocio, la alimentación, la higiene, el cambio de pañales, el sueño y el descanso e incluso cuidados específicos relacionados con la enfermedad^(64,95,103)^.

Sin embargo, hay situaciones en las que el padre no encuentra tiempo para implicarse en el cuidado del niño o no cree tener los conocimientos y habilidades necesarios para cuidar del niño enfermo. En estos casos, la madre asume el cuidado del niño enfermo. El principal factor de la falta de tiempo paterno está relacionado con las múltiples funciones que asumen los padres, en las que tienen que dividir su tiempo entre el trabajo remunerado para mantener a la familia, el cuidado de su hijo enfermo, el apoyo a su pareja, la responsabilidad de sus otros hijos y las tareas domésticas. De este modo, la participación paterna en el cuidado de un niño con una enfermedad grave es limitada^(88,103,104)^.

Con el fin de adquirir las habilidades necesarias para desempeñar su función paterna y cuidar de su hijo enfermo, los padres buscan información sobre la enfermedad, el tratamiento y el cuidado de la salud del niño en Internet, los libros y la televisión, y éste es un mecanismo que han encontrado para ayudar a su hijo. Cabe destacar que cuando un hombre proporciona los medios para satisfacer las necesidades psicoemocionales y físicas de su hijo, incluyendo la atención sanitaria, la alimentación, la higiene, la protección, el apoyo y el afecto, está cumpliendo una función denominada paternidad^(95,105,106)^.

El método canguro como estrategia para incluir a los padres en el cuidado de los niños hospitalizados en unidades de cuidados neonatales ha sido ampliamente discutido en la literatura. Los resultados muestran que la posición canguro refuerza el vínculo afectivo entre la díada padre-hijo, favorece una mayor participación paterna en el proceso de cuidado del neonato y da un nuevo significado a ser padre, ya que permite a los hombres vivir la paternidad de forma completa y significativa, conscientes de su papel como cuidadores. Al experimentar estos beneficios, el padre se siente más seguro en el cuidado del recién nacido hospitalizado. Esto despierta su deseo de permanecer más tiempo con su hijo^(107,108,109,110,111,112)^.

Cabe señalar que la unidad de cuidados intensivos neonatales es un entorno difícil para los padres. Para ellos, tener un hijo hospitalizado en este servicio sanitario significa la gravedad de la enfermedad y el riesgo de muerte. Los padres experimentan altos niveles de estrés, sufrimiento y miedo a perder a su hijo^(113)^.

Los estudios sobre el trastorno psicológico que sufren los padres ante la enfermedad grave de su hijo revelan experiencias dolorosas y aterradoras. Los padres se ven afectados por un intenso desequilibrio emocional que se expresa a través del sufrimiento, la tristeza, la ansiedad, la angustia, la desesperación, la desesperanza, la culpabilidad, el miedo a la muerte, la incertidumbre, la frustración y la incapacidad, lo que supone una condición preocupante, desafiante y estresante para todos los miembros de la familia^(114,115,116)^.

El padre también se ve afectado por la enfermedad, la hospitalización y el tratamiento de su hijo, experimentando miedo y temor ante la posibilidad de muerte del niño, frustración por tener un hijo enfermo con limitaciones, desesperación y tristeza por presenciar el estado de salud del niño e inutilidad por no poder aliviar el sufrimiento de su hijo. Sin embargo, los padres expresan sus sentimientos de forma diferente a las madres. Exteriorizan su dolor y preocupación llorando y mostrándose irritables y nerviosos, pero intentan no llorar delante de su mujer y familia, evitan hablar del problema y adoptan un comportamiento introspectivo, estando más callados de lo habitual^(51,67)^.

El diagnóstico de una enfermedad grave en un niño tiene repercusiones en términos de cambios en la dinámica y rutina familiar, adaptaciones en los roles parentales y restricciones en la vida social y laboral de los padres. La enfermedad y la hospitalización del niño causan perjuicios económicos a la unidad familiar debido a la reducción de las actividades laborales e incluso al abandono del trabajo, y la mayoría de las veces es la mujer la que abandona su vida profesional para dedicarse por completo a su hijo enfermo. Esta realidad se traduce en una mala calidad de vida y de ocio para el hombre, aislamiento social, excesiva carga de trabajo, falta de tiempo y prioridad para el autocuidado y sobrecarga de responsabilidades económicas para el padre. También hay un impacto negativo en las relaciones interpersonales familiares y conyugales^(117,118,119,120)^.

Ante la reorganización familiar para atender las necesidades del hijo enfermo, la unidad familiar se enfrenta a relaciones de distanciamiento y falta de convivencia entre sus miembros. La enfermedad del hijo es vista por los padres como un proceso perturbador, impregnado de infelicidad y estrés psicoemocional, que resulta en la ruptura de la identidad familiar y conyugal^(117,121,122)^.

Una vida conyugal saludable requiere equilibrio físico y psicológico para que pueda establecerse una relación íntima y amorosa entre la pareja, lo que no ocurre con los padres de niños enfermos, ya que la enfermedad del niño se coloca como principal foco de atención en la vida de los padres. Como consecuencia, la relación conyugal de los padres se ve mediatizada por la infelicidad, el estrés y la ruptura emocional. Además, la enfermedad del hijo provoca en los padres agotamiento físico y mental, pérdida de intimidad, desinterés sexual, conflictos y deterioro de la comunicación, contribuyendo al desgaste y a la separación conyugal^(123,124)^.

Considerando la importancia de resaltar la paternidad y estimular la inclusión de los padres en el cuidado de los niños con enfermedades graves, creemos estar colaborando de forma significativa con la comunidad académica, ya que la formación de los profesionales de salud y enfermería poco explora el cuidado centrado en la familia y hay desconocimiento sobre los padres. De esta forma, los resultados de esta revisión contribuyen a la docencia, a la investigación y a la construcción de un currículo integrado que incorpore temas sobre la unidad familiar y el rol paterno en sus contenidos de aprendizaje, con vistas a una formación profesional cualificada.

Este estudio también contribuye a la deconstrucción sociocultural de los ideales de género en relación con los roles parentales, al revelar el sufrimiento paterno en los casos en que la paternidad del hombre es dejada de lado en la vida de su hijo enfermo, los factores que contribuyen a que el padre no participe activamente en el cuidado de su hijo con una enfermedad grave y los beneficios que la presencia paterna promueve en la salud y el desarrollo del niño, en el bienestar materno y en los cuidados prestados por los profesionales de enfermería.

Las limitaciones de esta revisión exploratoria incluyen la elección de estudios sólo en inglés, español y portugués, la inclusión de sólo las diez primeras páginas de Google Scholar y la no disponibilidad del texto completo de algunos artículos, lo que puede haber dificultado la recopilación de datos y la selección de estudios relevantes. Otro factor limitante fue el contexto de la investigación, ya que los temas del cuidado paterno y la igualdad de género son poco tratados en la literatura científica, especialmente cuando se asocian a padres que cuidan de sus hijos con enfermedades graves.

## CONCLUSIÓN

Los resultados de esta revisión permitieron mapear y describir la participación paterna en el cuidado de un hijo con una enfermedad grave. Este estudio encontró que la participación de los padres en el cuidado de sus hijos con enfermedades graves es limitada. Incluso frente a los beneficios de la participación paterna en el cuidado del niño enfermo, los padres se enfrentan a obstáculos para ejercer su paternidad activa relacionados con cuestiones socioculturales y de género relativas a los roles parentales. Además, se constató que ser padre de un niño con enfermedad grave tiene repercusiones negativas en la vida del hombre, causando impactos económicos, familiares y psicoemocionales.

Se identificaron lagunas en la formación académica de los profesionales de la salud en paternidad y atención centrada en la familia, destacando la importancia de la capacitación, incluso durante la graduación, para trabajar en la atención a la salud infantil, ya que muchos profesionales se enfrentan a estigmas históricos, socioculturales y de género relacionados con los roles parentales, lo que resulta en una atención inhumana y fragmentada para la tríada madre, padre e hijo y, como consecuencia, limita el ejercicio de la paternidad.

Por último, cabe decir que es necesario seguir investigando para indagar en cuestiones que aún no han sido suficientemente exploradas y con las mejoras metodológicas necesarias para avanzar en la comprensión de las repercusiones socioculturales y de género que influyen y limitan el ejercicio de la paternidad, el rol paterno y los beneficios de la presencia paterna y la inclusión de los padres en el cuidado de los niños con enfermedades graves.

Además, los estudios futuros deben tener como objetivo proporcionar a los profesionales de la salud, investigadores y responsables de las políticas públicas un conocimiento vertical sobre la paternidad, con el fin de apoyar la incorporación de estrategias en la práctica asistencial y en los servicios de salud infantil y adolescente para mejorar la experiencia paterna del cuidado de un niño con una enfermedad grave, implicar a la figura paterna en las relaciones familiares, intensificar la humanización de los cuidados a la unidad familiar, promoviendo acciones que fortalezcan el vínculo paterno-filial y revelen una paternidad activa.
